# Proanthocyanidin B2 Alleviates Cuprizone‐Induced Demyelination by Regulating the Astrocytic xCT/GSH/GPX4 Axis

**DOI:** 10.1111/cns.70598

**Published:** 2025-09-18

**Authors:** Jian Liu, Yan‐Xia Hou, Ying Chen, Ya‐Jie Liang, Meng Pu, Lu‐Lu Zheng, Zi‐Wei Zhang, Ying Xiao, Zhen Mao, Cun‐Gen Ma, Qing Wang

**Affiliations:** ^1^ Research Center of Neurobiology, the Key Research Laboratory of Benefiting Qi for Acting Blood Circulation Method to Treat Multiple Sclerosis of State Administration of Traditional Chinese Medicine Shanxi University of Chinese Medicine Jinzhong Shanxi China; ^2^ Caidian District People's Hospital Wuhan Hubei China

**Keywords:** antioxidants, astrocyte, cuprizone, demyelination, proanthocyanidin B2, xCT/GSH/GPX4 axis

## Abstract

**Background:**

Multiple sclerosis (MS) is marked by inflammatory demyelination in the central nervous system (CNS), involving diverse glial populations. This pathological process is associated with inflammation and oxidative stress. Proanthocyanidin B2 (PCB2), with its potent antioxidant properties, has been shown to alleviate demyelination in the cuprizone (CPZ) mouse model. It attenuates neuroinflammation and oxidative stress in both the cerebral microenvironment and astrocytes (AS). The xCT/GSH/GPX4 axis is a key regulatory pathway for oxidative stress. Therefore, we hypothesize that PCB2 can alleviate CPZ‐induced demyelination by regulating the xCT/GSH/GPX4 axis in AS.

**Methods:**

The study utilized forty C57BL/6 mice, randomly allocated into four groups of ten: a control group, a control group supplemented with PCB2 (60 mg/kg/day), a CPZ‐exposed group, and a CPZ‐exposed group supplemented with PCB2 (60 mg/kg/day). The control groups received a standard diet, whereas the CPZ groups were given the same diet supplemented with 0.2% CPZ for 6 weeks. From the fifth week onwards, the control and CPZ groups were administered physiological saline via intraperitoneal injection, whereas the PCB2‐supplemented groups received PCB2 for 2 weeks. Immunofluorescence staining, Western blot, and ELISA elucidated the cellular/molecular mechanisms of PCB2 targeting the xCT/GSH/GPX4 axis in AS to alleviate demyelination in vivo and in vitro.

**Results:**

In this study, PCB2 markedly regulated the xCT/GSH/GPX4 axis in AS, ameliorated the behavioral performance in CPZ mice, reduced inflammation, oxidative stress, lipid peroxidation, and the damage to oligodendrocytes (OLs), and inhibited demyelination.

**Conclusion:**

PCB2 can regulate the entire xCT/GSH/GPX4 axis of AS to reduce CPZ‐induced OL injury and demyelination, which may be a potentially effective drug for the treatment of multiple sclerosis.

## Graphical abstract

Proanthocyanidin B2 alleviates cuprizone‐induced demyelination in mice by modulating the astrocytic xCT/GSH/GPX4 axis, reducing inflammation and oxidative stress, and protecting oligodendrocytes, offering a novel therapeutic strategy for multiple sclerosis.

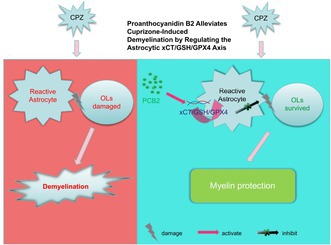

## 1 Introduction

MS is an inflammatory demyelinating disease of the CNS hallmarked by a neuroinflammatory response, oxidative stress, and myelin loss [1]. Clinical manifestations include motor, cognitive, and sensory dysfunction [2]. MS tends to occur in young people, with about 2.5 million patients worldwide, and the incidence rate is increasing year by year. The pathogenesis of MS is complex, and its potential pathological mechanisms include neuroinflammation, loss of mature OLs, axonal injury, oxidative stress, and an increase in microglia (MG) [3]. At present, the treatment strategies for MS include the use of immunosuppression, immune modification, and symptomatic support, but these drugs and treatments have limited efficacy and high medical costs. Therefore, studying the pathogenesis of MS and searching for new therapeutic drugs have important clinical significance [4].

The cystine/glutamate transporter (xCT)/glutathione (GSH)/glutathione peroxidase (GPX4) axis is a key oxidative stress regulatory axis that plays important antioxidant and anti‐inflammatory roles in the body. Among them, xCT is a cysteine/glutamate transporter responsible for transporting cysteine (Cys) into cells, providing precursors for GSH synthesis. Glutathione (GSH) is an important intracellular antioxidant that can clear Reactive Oxygen Species (ROS) and Reactive Nitrogen Species (RNS). The xCT/GSH/GPX4 axis regulates oxidative stress by maintaining GSH levels, which is also a key target for inhibiting cell ferroptosis [5]. GPX4 is a GSH dependent peroxidase that catalyzes the reduction of phospholipid hydroperoxides and hydrogen peroxide, thereby protecting cell membranes from oxidative damage. In terms of inflammation, the xCT/GSH/GPX4 axis helps reduce oxidative stress by maintaining GSH levels [6]. Thus, it inhibits the production of inflammatory mediators, such as cytokines and chemokines, and breaks the positive feedback loop between oxidative stress and inflammation. This helps to alleviate inflammatory reactions and protect cells from damage. In neurodegenerative diseases such as MS, oxidative stress and inflammation are important pathological factors. Regulating the xCT/GSH/GPX4 axis may help with antioxidant stress and anti‐inflammatory effects, thereby protecting nerve cells [7].

AS is the most abundant non‐neuronal cell in the brain, playing an important role in CNS development and plasticity. It can control synaptic transmission, regulate blood flow, energy, and metabolism, participate in the formation of the blood–brain barrier (BBB), regulate lipid metabolism, and nerve regeneration [8, 9]. In an inflammatory and/or demyelinating environment, reactive astrocytes (RAs) proliferate extensively and their classic marker, glial fibrillary acidic protein (GFAP), is upregulated [10]. Meanwhile, as the parenchymal cells involved in the formation of the BBB, AS is the first line of defense against exogenous oxidative damage and has its own antioxidant defense function. When it is activated, on one hand, AS can recruit immune cells to the lesion site, produce inflammation, and cause tissue damage; on the other hand, it can also inhibit excessive inflammation and promote neuroprotection and repair [11, 12], and its effect is more long‐lasting than MG. Therefore, regulating AS has become an important therapeutic strategy for demyelinating diseases such as MS.

CPZ is a copper chelator that can cause CNS demyelination, especially in the corpus callosum (CC), and is therefore widely used as a model for studying demyelinating diseases (such as MS). PCB2 is a natural flavonoid substance belonging to the B‐type anthocyanin dimer and has been proven to have stronger antioxidant, anti‐inflammatory, and anti‐tumor effects than other anthocyanins such as B1, B4, and B5 [13, 14, 15], which can alleviate oxidative stress‐induced damage to the CNS, inhibit inflammatory responses, and alleviate inflammatory damage in neurological diseases [16]. It can also inhibit oxidative damage to glial cells and protect the integrity of myelin sheath [17]. Furthermore, as a drug that can alleviate oxidative stress in CNS cells, PCB2 has been shown to regulate the protein expression level of GPX4 in the brain [18]. Therefore, there may be a wider range of applications in the treatment of CNS diseases. However, the protective effect of PCB2 on the CNS myelin sheath and its molecular mechanism need further clarification. So, in a mouse demyelination model, can PCB2 regulate the xCT/GSH/GPX4 axis of AS with its excellent antioxidant capacity and myelin protection, thereby inhibiting CPZ‐induced OLs injury and myelin loss? This will be the purpose of this study.

## 2 Material and Method

### 2.1 Animals and Cells

Forty male C57BL/6 mice, aged 7–8 weeks and weighing 20–22 g, were obtained from Beijing Weitong Lihua Experimental Animal Co. Ltd., licensed under SCXK (Beijing) 2016–0006. These mice were housed in a specific pathogen‐free (SPF) facility at Shanxi University of Chinese Medicine, where they were allowed to acclimate for 1 week before experimentation. Additionally, 24–48 h neonatal C57BL/6 mice were sourced from the Experimental Animal Center of Shanxi Medical University, licensed under SCXK (Jin) 2019–004. Primary OLs were obtained from Shanghai Fusheng Industrial Co. Ltd., Primary OLs basic culture medium (Shanghai Fusheng Industrial Co. Ltd., A01XZ398, China).

### 2.2 Methods

#### 2.2.1 Establishment of the CPZ Animal Model

The study utilized forty C57BL/6 mice, randomly assigned into four groups of ten: control group, control group supplemented with PCB2, CPZ‐exposed group, and CPZ‐exposed group supplemented with PCB2. The control groups received a standard diet, whereas the CPZ groups were given the same diet supplemented with 0.2% CPZ for 6 weeks. From the fifth week onwards, the control and CPZ groups were administered physiological saline via intraperitoneal injection, whereas the PCB2‐supplemented groups received PCB2 (Chengdu Rufens Biological Technology Co. Ltd., purity ≥ 98%, 60 mg/kg/day) for 2 weeks.

#### 2.2.2 Primary AS Isolation and Recovery Experiment Model Establishment

Homogenates were extracted from the brains of 10 C57BL/6 newborn mice aged 1–2 days, filtered, and subjected to a differential adhesion culture method for purification. Cultured in complete medium, 94% inoculation medium (gibco, 12,430–054, USA), 5% fetal bovine serum (gibco, 10,091–148, USA) and 1% Penicillin–Streptomycin Solution (Solarbio, P1400, China). The purity of the AS was confirmed through GFAP immunostaining. AS was then treated with TNF‐α (30 ng/mL), IL‐1α (3 ng/mL), Complement C1q (400 ng/mL), RSL3 (a GPX4 inhibitor), PCB2, and Erastin (an xCT inhibitor) for 24 h to create a model of RAs for recovery experiments and their respective inhibitory models. The conditioned medium from the cultured RAs, aged 24–48 h, was applied to OLs cultures for an additional 24 h.

#### 2.2.3 Behavioral Assessment

Following the treatment regimen, a comprehensive behavioral assessment was performed. Behavioral assessment included: open field test (OFT) to measure general locomotor activity and anxiety‐like behavior, the elevated plus maze (EPM) to assess anxiety and spatial memory, the climbing pole test (CPT) to evaluate motor coordination and strength, and the Y‐maze to test spatial working memory.

#### 2.2.4 Sample Collection and Preparation

Animal Tissue Collection: Following the completion of behavioral experiments, four mice from each group were randomly selected and anesthetized with pentobarbital sodium (50 mg/kg). The chest was opened to expose the heart, and the right auricle was cut to perfuse with physiological saline until the liver turned pale, followed by perfusion with 4% paraformaldehyde. The brain tissue was then dissected, fixed with paraformaldehyde, and subjected to a gradient dehydration series in 10%, 20%, and 30% sucrose solutions. Subsequently, the tissue was embedded in OCT compound, flash‐frozen in liquid nitrogen, and stored at −80°C for future use. When required, the tissue was sectioned into 10 μm slices using a cryostat for immunofluorescence staining. In addition, three mice per group were perfused with saline, after which the cerebrum was isolated. The tissue fragments were washed three times with phosphate buffered saline (PBS) and centrifuged for cell lysis; the remaining tissue was ground, lysed, centrifuged, and the supernatant was collected in sterile EP tubes for protein content measurement and long‐term storage at −80°C. The cerebra from the remaining three mice were processed to isolate myelin fragments via ultracentrifugation [19], then concentrated and stored at −80°C.

Cell Sample Collection: For the cell cultures, the supernatant from AS cultures was discarded, and the cells were harvested, lysed, resuspended, and centrifuged. The resulting supernatant was transferred to fresh sterile EP tubes, where protein concentration was determined, aliquoted, and stored at −80°C.

#### 2.2.5 Luxol Fast Blue (LFB) and TrueGold Staining Detection

For Luxol Fast Blue staining (Solarbio, G3245, China), mouse brain sections were initially warmed and soaked in 70% ethanol for 15 min before being incubated in the staining solution at 56°C–60°C overnight. The next day, sections were thoroughly rinsed with 95% ethanol and deionized water to remove surplus stain, then treated with a mixture of 75% ethanol, deionized water, and 0.05% lithium carbonate to clearly differentiate gray and white matter. Following a series of ethanol dehydration steps and xylene clearing, the sections were mounted with neutral resin for microscopic examination.

For TrueGold staining (Ousaisi Biotechnology Co. Ltd., BK‐AC001, China), brain slices were warmed and dried at 37°C for 30 min before diluting the stain and terminator in double‐distilled water. The slices were then stained in the dark at 45°C for 30 min, rinsed with PBS, and treated with the stain terminator at 45°C for 2–3 min. After three washes with PBS, the slices were dried, mounted, and imaged using a microscope.

#### 2.2.6 Immunofluorescence Staining

Prepared frozen sections of mouse brain tissue were washed three times for 5 min each with PBS; the primary antibody was added to a PBS solution containing 0.3% Triton X‐100 and 1% BSA, mixed thoroughly, and then applied to the sections for overnight incubation at 4°C in a humid chamber. The following day, the sections were washed three more times with PBS for 5 min each, and then incubated with the corresponding secondary antibody at room temperature for 1 h. After three final washes with PBS, the slides were mounted using a fluorescence‐quenching mounting medium containing DAPI. Protein expression was assessed: Myelin Basic Protein (MBP, 1:2000 dilution, Abcam, ab40390, Britain;1:250 dilution, Bio‐Rad, aa82‐87, USA), Degenerate Myelin Basic Protein (dMBP, 1:1000 dilution, sigma, AB5864, Germany), Neuroglial Antigen 2 (NG2, IF 1:500 dilution, Millipore, 05–710, USA), Glial Fibrillary Acidic Protein (GFAP, 1:1000 dilution, Oasis Biofarm, OB‐PGP055, China; IF 1:1000 dilution, thermofisher, PA1‐10004, USA), Ionized Calcium Binding Adapter Molecular 1 (IBA1, 1:100 dilution, huabio, ET1705‐78, China), Nuclear Receptor Coactivator 4 (NCOA4, 1:250 dilution, Affinity, DF4255, USA), Ferritin (IF 1:100 dilution, huabio, ET1610‐78, China), Nuclear Factor erythroid 2‐related factor 2 (NRF2, 1:200 dilution, ZEN BIO,380773, USA), GPX4 (IF 1:1000 dilution, Proteintech, 67,763–1‐Ig, China), and System xc‐ (Cystine‐Glutamate Antiporter)/Solute Carrier Family 7 Member 11 (xCT/SLC7A11, IF 1:200 dilution, huabio, HA601071, China) in the mouse brain CC region under a fluorescence microscope. The fluorescence intensity was analyzed using Image J fiji 2.14.0 software.

#### 2.2.7 Enzyme‐Linked Immunosorbent Assay (ELISA) and Enzyme Assay Detection

Collect brain protein extracts, myelin fragments, and cell samples from each group, and operate according to the instructions of the ELISA and enzyme assay. Use an enzyme labeler to measure the optical density (OD) values, which will help in determining the levels or activity of various proteins and enzymes in the samples. Specifically, such as Interleukin‐1 Beta (IL‐1β, Invitrogen, 88‐7013A‐88, USA), Interleukin‐6 (IL‐6, Invitrogen, 88–7064‐22, USA), Interleukin‐10 (IL‐10, Invitrogen, 88–7105‐22, USA), Tumor Necrosis Factor‐Alpha (TNF‐α, Invitrogen, 88–7324‐88, USA), Catalase (CAT, Pulilai Gene Technology Co. Ltd., E2030, China), Superoxide dismutase (SOD, Nanjing Jiancheng, A003‐4‐1, China), Nitric oxide (NO, Beijing Pulilai Gene Technology Co. Ltd., E1030, China), Glutathione peroxidase (GSH‐Px, Nanjing Jiancheng, A005‐1‐2, China), Lipid peroxide (LPO, Nanjing Jiancheng, A106‐1, China), Glutathione (GSH, Nanjing Jiancheng, A061‐1, China), TNF‐α, hydrogen peroxide (H_2_O_2_, Nanjing Jiancheng, A064‐1‐1, China) in the mouse brain homogenate. Additionally, the content of CAT, SOD, Malondialdehyde (MDA, Beijing Pulilai Gene Technology Co. Ltd., E2019, China), and LPO will be assessed in the myelin fragments [20], while GSH, Glutamine (Glu, Cell Biolabs, CBL‐MET‐5166, China) levels will be evaluated in AS samples.

This step involves using specific assay kits to quantify or assess the biological activity of various markers and enzymes in the brain tissue samples. The OD values obtained from the enzyme labeler are directly proportional to the concentration of the target analytes, allowing for the assessment of inflammation, oxidative stress, and other biological processes within the brain tissue.

#### 2.2.8 Western Blot Detection

Utilizing the aforementioned brain homogenate proteins and the lysed proteins from AS groups in the inhibition experiments, the BCA (Bicinchoninic Acid) method is employed to determine the concentration of the extracted protein samples. A quantitative 30 μg of each sample is prepared for loading. The protein samples are added to the Loading Buffer and boiled for 10 min for denaturation before being set aside. After gel preparation based on the molecular weight of the proteins, the proteins are wet‐transferred onto an activated PVDF (Polyvinylidene Fluoride) membrane via SDS‐PAGE (Sodium Dodecyl Sulfate‐Polyacrylamide Gel Electrophoresis). A 5% BSA (Bovine Serum Albumin) blocking solution is used for blocking at room temperature for 2 h. Following TBST (Tris‐Buffered Saline with Tween) washing, the primary antibody is added and incubated overnight in a 4°C refrigerator. The next day, TBST is used to rinse away the excess primary antibody three times, and then the secondary antibody is added and incubated on a shaker at room temperature for 2 h. After three more TBST rinses, the ECL (Enhanced Chemiluminescence) reagent is applied, and the protein expression bands for NCOA4 (WB 1:1000 dilution, Affinity, DF4255, USA), Ferritin (1:1000 dilution, huabio, ET1610‐78, China), NRF2 (1:2500 dilution, Proteintech,80,593–1‐RR, China), GPX4 (1:1500 dilution, Proteintech, 67,763–1‐Ig, China), and xCT (1:3000 dilution, huabio,HA601071, China) in the brain homogenate are visualized using a gel imaging system. The gray values of the bands are analyzed using Image J software.

#### 2.2.9 Molecular Docking and Molecular Dynamics Simulation (MD)

Query the key target gene protein data bank (PDB) ID using the UniProt database, and download the corresponding protein molecule (receptor) 3D structure of the target using the RCSB PDB website (xCT/SLC7A11, PDB ID: 7EPZ/GPX4, PDB ID: 5H5Q). Download the 3D structure of PCB2 (ligand) from the PubChem database (PCB2, Chemical Abstracts Service (CAS) NO: 29106–49‐8), perform molecular docking using AutoDockTools‐1.5.7, calculate the free energy, and then use PyMOL Molecular Graphics System Version 4.6.0 to draw the optimal docking diagram.

MD were performed using Gromacs2022. The small molecule was modeled with the GAFF force field, and the protein with the AMBER14SB force field combined with the TIP3P water model. The simulations were conducted under constant temperature and pressure with periodic boundary conditions. Hydrogen bonds were constrained using the LINCS algorithm, with an integration step of 2 fs. Electrostatic interactions were calculated via the PME method with a cutoff of 1.2 nm, and non‐bonded interactions had a cutoff of 10 Å, updated every 10 steps. The system was maintained at 298 K using the V‐rescale method and at 1 bar pressure using the Berendsen method. After 100 ps of NVT and NPT equilibration, a 100 ns production simulation was carried out, with snapshots saved every 10 ps for analysis using VMD and PyMol.

After the simulation was completed, the root mean square deviation (RMSD), root mean square fluctuation (RMSF), and protein radius of gyration (Rg) of each amino acid trajectory was calculated by using Python 3.8.5 combined with the free energy (MMGBSA), the Free—energy landscape analysis, and other data.

#### 2.2.10 Cell Viability and Cytotoxicity Assessment

Cell Counting Kit‐8 (CCK‐8) Assay, and cell viability were assessed using the CCK‐8 assay (Beyotime Biotechnology, C0038, China). Cells were plated in 96‐well plates at a density of 7500 to 10,000 cells per well and incubated overnight. After medication treatment for 24 h, 10 μL of the CCK‐8 solution was added to each well, and the plates were incubated at 37°C for 1 h in a humidified atmosphere containing 5% CO_2_. The absorbance at 450 nm was measured using a microplate reader. The viability of the cells was calculated as a percentage of the absorbance of the untreated control cells.

L‐Lactate Dehydrogenase (L‐LDH) Assay, and cell cytotoxicity was evaluated using an L‐LDH assay kit (Solarbio, bc0685, China) according to the manufacturer's instructions. Cells were plated in 96‐well plates at a density of 7500 to 10,000 cells per well and incubated overnight. After medication treatment for 24 h, the culture supernatant (180 μL) was collected from each well and, according to the steps in the manual, the absorbance was measured at 450 nm using a microplate reader. Compare the OD values of each group.

We used CCK‐8 and L‐LDH assays to screen for the optimal drug concentrations of PCB2, RSL3, and Erastin. To assess the effects of AS supernatants from different groups on OLs, we used the supernatant after a 24–48 h culture period and applied it as a conditioned medium for OLs for an additional 24 h. We then collected the supernatant from each group and utilized the CCK‐8 and L‐LDH assays to evaluate their impact on OLs.

#### 2.2.11 Ferrous Ion Fluorescent Probe (FerroOrange) Detection of Intracellular Ferrous Ions (Fe^2+^) Content

Inoculate each cell of the group into confocal culture dishes and culture overnight in a 37°C incubator with 5% CO_2_. After the cells have adhered, administer the medication according to the selected dosage groups and continue to culture for 24 h. Remove the culture medium and wash with PBS three times. Add the working solution of FerroOrange (DOJINDO, F374, Japan) at a concentration of 1 mol/L and culture in a 37°C incubator with 5% CO_2_ for 30 min. There is no need for further washing; observe directly under a laser confocal microscope after the culture. Analyze the fluorescence intensity using ImageJ Fiji 2.14.0 software.

#### 2.2.12 AI‐Assisted Language Polishing

In addition to the above experimental methods, we employed AI‐generated content tools for language polishing to enhance the clarity and readability of our manuscript. Specifically, we utilized Deepseek for refining the language and grammar without altering the scientific content or conclusions drawn from our research. This tool was particularly useful in ensuring that our manuscript met the high standards of scientific communication.

#### 2.2.13 Statistical Analysis

Statistical analysis was performed using GraphPad Prism 8.0.2 software. For all datasets, normality was assessed using the Shapiro–Wilk test. Data that conform to a normal distribution are represented as $\bar{x}$ ± SD. For comparisons among multiple groups, one‐way ANOVA analysis was used, and for pairwise comparisons between groups, Tukey's post hoc test was employed. For non‐normally distributed data, the Mann–Whitney *U* test and Kruskal–Wallis test were applied. A *p*‐value of less than 0.05 was considered to indicate a statistically significant difference.

## 3 Results

### 3.1 Effects of PCB2 on Behavioral Outcomes in CPZ‐Treated Mice

**FIGURE 1:**
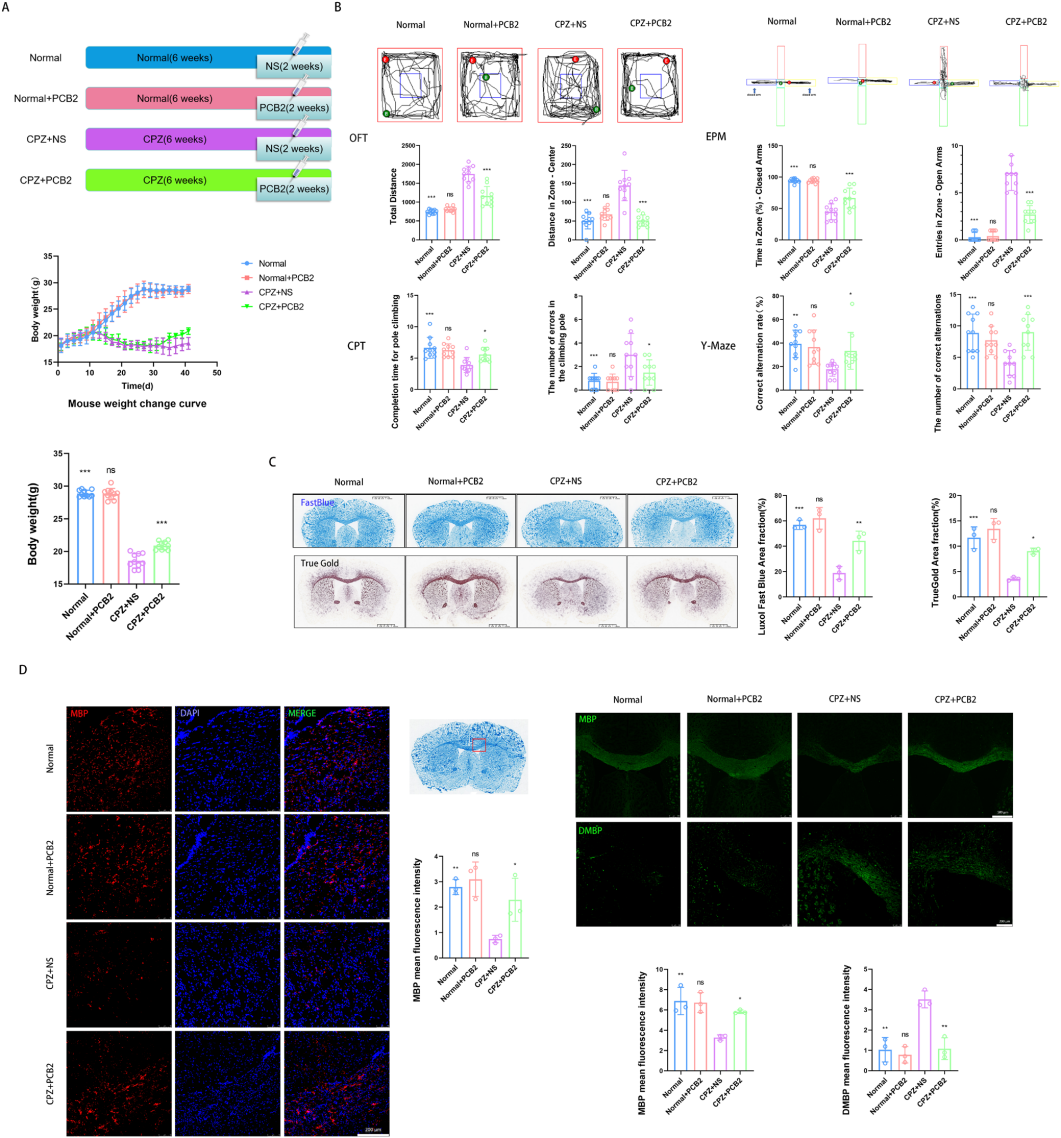
Effects of PCB2 on Mouse Weight, Behavior, and Histology in CPZ mice. (A) The effect of PCB2 on the weight changes of mice in each group during modeling and treatment, as well as the effect on weight at the end of 6 weeks ($\bar{x}$ ± SD, *n* = 10). ***p < 0.001, comparison between Normal + PCB2 group and Normal group, comparison between other groups and CPZ + NS group (two‐way analysis of variance with Tukey's post hoc test). (B) The effect of PCB2 on behavioral performance (OFT, EPM, CPT and Y‐Maze) of CPZ mice ($\bar{x}$ ± SD, *n* = 10). *p < 0.05, **p < 0.01, ***p < 0.001, comparison between Normal+PCB2 group and Normal group, comparison between other groups and CPZ + NS group (two‐way analysis of variance with Tukey's post hoc test). (C) The effect of PCB2 on Histological Staining with LFB (Scale Bar = 1.25 mm, $\bar{x}$ ± SD, *n* = 3) and TrueGold (Scale Bar = 1.25 mm, $\bar{x}$ ± SD, *n* = 3). *p < 0.05, **p < 0.01, ***p < 0.001, comparison between Normal+PCB2 group and Normal group, comparison between other groups and CPZ + NS group (two‐way analysis of variance with Tukey's post hoc test). (D) The effect of PCB2 on Immunofluorescence Staining for MBP (red, Magnification 20×, Scale Bar = 200 μm, $\bar{x}$ ± SD, *n* = 3; green, Magnification 5×, Scale Bar = 500 μm, $\bar{x}$ ± SD, *n* = 3), and dMBP (green, Magnification 10×, Scale Bar = 200 μm, $\bar{x}$ ± SD, *n* = 3) of CPZ mice. *p < 0.05, **p < 0.01, comparison between Normal+PCB2 group and Normal group, comparison between other groups and CPZ + NS group (two‐way analysis of variance with Tukey's post hoc test).

During the experimental procedure, we closely monitored the body weight of mice across all groups. As depicted in Figure 1A, a notable decrease in body weight was observed in both CPZ + NS and CPZ + PCB2 groups from the 12th day post‐modeling, with a significant reduction by the sixth week's end (*p <* 0.001). Notably, the CPZ + PCB2 group demonstrated a significant recovery in body weight during the final weeks of the treatment compared to the CPZ + NS group (*p <* 0.001).

Behavioral Assessments: Given the potential for cognitive and behavioral disturbances in the CPZ mouse model, including memory impairments and anxiety, we evaluated the impact of PCB2 on these parameters. As shown in Figure 1B, in the OFT, the CPZ + NS group exhibited an increase in total movement and central zone activity (*p <* 0.001), indicative of hyperactivity and anxiety. In contrast, the CPZ + PCB2 group showed a reduction in these measures (*p <* 0.001), suggesting a mitigating effect of PCB2 on CPZ‐induced behavioral changes.

The EPM was utilized to assess anxiety‐related behaviors, revealing that the CPZ + NS group spent less time on the closed arms and made more entries into the open arms (*p <* 0.001), a behavior associated with heightened anxiety. The CPZ + PCB2 group, however, spent more time on the closed arms and made fewer open arm entries (*p <* 0.001 and *p <* 0.001, respectively), indicating a potential anxiolytic effect of PCB2.

In the CPT, which measures motor coordination and caution, the CPZ + NS group displayed quicker descent times and increased errors (*p <* 0.001), suggesting impaired motor function. Conversely, the CPZ + PCB2 group showed improved performance, with increased time to descend and fewer mistakes (*p <* 0.05), indicative of PCB2's neuroprotective potential.

In the Y‐Maze test, assessing spatial learning and memory, revealed a decrease in correct alternations and alternation rate in the CPZ + NS group (*p <* 0.001 and *p <* 0.01, respectively). In contrast, the CPZ + PCB2 group exhibited an increase in these parameters (*p <* 0.001 and *p <* 0.05, respectively), suggesting that PCB2 may enhance spatial learning and memory in the context of demyelination.

### 3.2 PCB2 Ameliorates Demyelination in CPZ‐Treated Mice

#### 3.2.1 Histological Assessments

As illustrated in Figure 1C, LFB and TrueGold staining revealed a significant decrease in myelin staining within the CC region of the CPZ + NS group compared to the control group (both *p <* 0.001). Notably, the CPZ + PCB2 group exhibited a significant increase in myelin staining, indicative of PCB2's neuroprotective effects (*p <* 0.01 for LFB, *p <* 0.05 for TrueGold).

#### 3.2.2 Immunofluorescence Analysis

The immunofluorescence staining within the CC region depicted in Figure 1D demonstrated a significant reduction in MBP expression (Two different antibodies and amplification factors) and a corresponding increase in dMBP expression in the CPZ + NS group, suggesting compromised myelin integrity (both *p <* 0.01). In stark contrast, the CPZ + PCB2 group showed a significant upregulation of MBP and a decrease in dMBP, reflecting a denser myelin structure and the protective influence of PCB2 (*p <* 0.05 for MBP, *p <* 0.01 for dMBP).

### 3.3 Anti‐Inflammatory and Antioxidant Effects of PCB2 in CPZ‐Treated Mice

**FIGURE 2:**
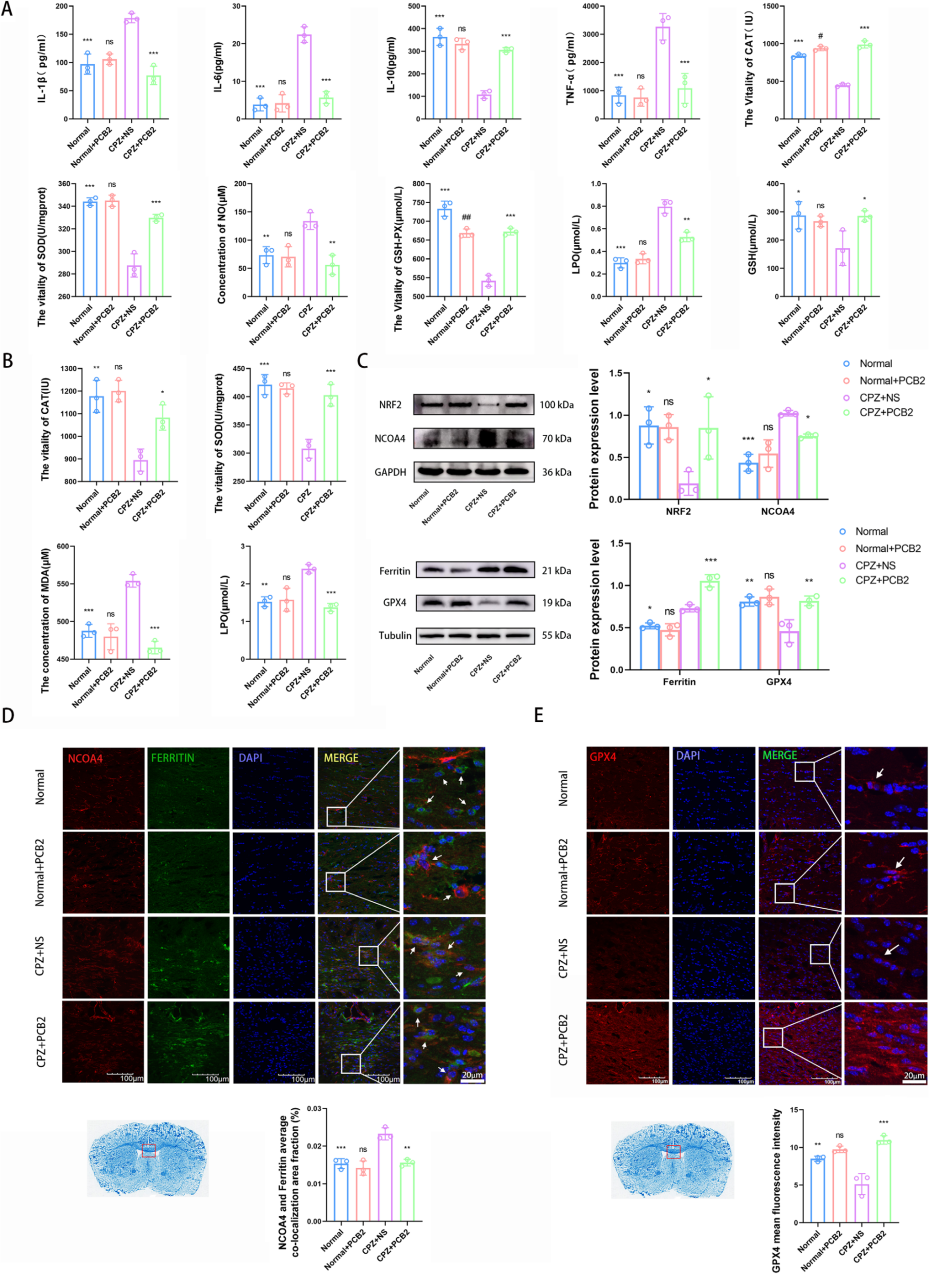
Impact of PCB2 on Inflammatory and Oxidative Stress Responses in CPZ mice. (A) ELISA and enzyme assay show the effect of PCB2 on inflammatory and oxidative stress responses in the whole brain of CPZ mice ($\bar{x}$ ± SD, *n* = 3). *p < 0.05, **p < 0.01, ***p < 0.001, compared to CPZ + NS Group, ^#^ *p <* 0.05, ^##^ *p <* 0.01 compared to the Normal group (two‐way analysis of variance with Tukey's post hoc test). (B) ELISA and enzyme assay show the effect of PCB2 on inflammatory and oxidative stress responses in the myelin debris of CPZ mice ($\bar{x}$ ± SD, *n* = 3). *p < 0.05, **p < 0.01, ***p < 0.001, comparison between Normal + PCB2 group and Normal group, comparison between other groups and CPZ + NS group (two‐way analysis of variance with Tukey's post hoc test). (C) Western blot detection results show the effect of PCB2 on the protein expression level of NCOA4, NRF2, Ferritin, and GPX4 in the brain homogenate of CPZ mice ($\bar{x}$ ± SD, *n* = 3). *p < 0.05, **p < 0.01, ***p < 0.001, comparison between Normal+PCB2 group and Normal group, comparison between other groups and CPZ + NS group (two‐way analysis of variance with Tukey's post hoc test). (D) Immunofluorescence staining shows the effect of PCB2 on the expression of NCOA4 (red) and Ferritin (green) average co‐localization area fraction (%) in the CC region of CPZ mice (Magnification 40×, Scale Bar = 100 μm, $\bar{x}$±SD, *n* = 3). **p < 0.01, ***p < 0.001, comparison between Normal+PCB2 group and Normal group, comparison between other groups and CPZ + NS group (two‐way analysis of variance with Tukey's post hoc test). (E) Immunofluorescence staining shows the effect of PCB2 on the expression of GPX4 (red) in the CC region of CPZ mice (Magnification 40×, Scale Bar = 100 μm, $\bar{x}$ ± SD, *n* = 3). **p < 0.01, ***p < 0.001, comparison between Normal+PCB2 group and Normal group, comparison between other groups and CPZ + NS group (two‐way analysis of variance with Tukey's post hoc test).

As shown in Figure 2A, the CPZ + NS group displayed significantly elevated levels of pro‐inflammatory cytokines (IL‐1β, IL‐6, TNF‐α) and oxidative stress indicators (NO, LPO) compared to the control group (all *p <* 0.001, except where noted). Treatment with PCB2 led to a significant reduction in these levels, indicative of its anti‐inflammatory and antioxidant properties (all *p <* 0.01, except where noted). Conversely, the CPZ + NS group showed decreased levels of the anti‐inflammatory cytokine IL‐10 and the antioxidant enzyme activities of CAT, SOD, and GSH‐Px, which were restored to near normal upon PCB2 treatment.

Further analysis of myelin fragments, isolated by ultracentrifugation, revealed that CPZ + PCB2 treatment significantly increased the activities of CAT and SOD and decreased the levels of MDA and LPO, suggesting a protective role of PCB2 in mitigating oxidative stress within the myelin sheath (Figure 2B).

In order to understand the expression of lipid peroxidation related proteins, we conducted Western blot experiments. Western blot analysis of brain homogenates revealed that compared to the CPZ + NS group, the PCB2 treatment group significantly elevated the expression of NRF2, GPX4, and Ferritin (*p <* 0.05, *p <* 0.01, *p <* 0.001, respectively), while decreasing NCOA4 levels (*p <* 0.05), further supporting the antioxidant and anti‐inflammatory effects of PCB2 (Figure 2C). These changes in protein expression suggest a potential mechanism by which PCB2 modulates the response to oxidative stress and inflammation in CPZ mice.

After that, the suppressive effect of PCB2 on lipid peroxidation was substantiated through immunofluorescence analysis of the co‐localization of NCOA4 and Ferritin in the CC region. A notably decreased co‐localization area fraction was observed in the CPZ + PCB2 group relative to the CPZ + NS group (*p <* 0.01, Figure 2D). Furthermore, GPX4, essential for antioxidant defense, exhibited diminished fluorescence intensity within the CPZ + NS group, which was significantly countered by treatment with PCB2 (*p <* 0.001, Figure 2E).

### 3.4 Upregulation of GPX4 Expression in AS Within the CC Region by PCB2

Immunofluorescence assays were performed to assess the influence of PCB2 on the expression of GPX4 in different cell types within the CC region of CPZ mice.

**FIGURE 3:**
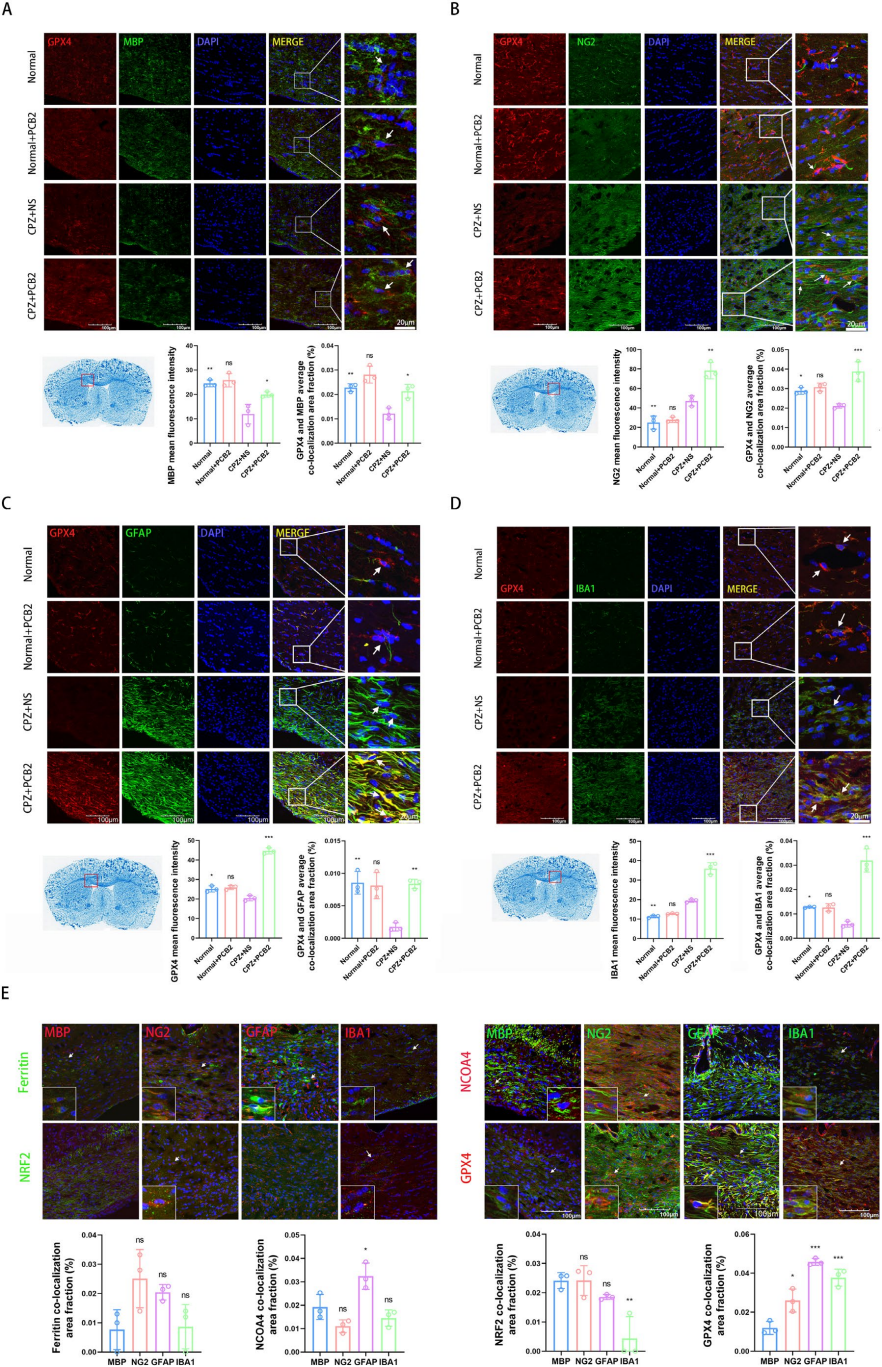
Immunofluorescence Analysis of PCB2's Impact on co‐localization with OLs, OPCs, AS, and MG in the CC region of CPZ Mice. (A) Immunofluorescence results indicate the effect of PCB2 on the expression MBP (green) in the CC region of CPZ mice, along with the average co‐localization area fraction (%) of GPX4 (red) in MBP+ (Magnification 40×, Scale Bar = 100 μm, $\bar{x}$±SD, *n* = 3). *p < 0.05, **p < 0.01, comparison between Normal+PCB2 group and Normal group, comparison between other groups and CPZ + NS group (two‐way analysis of variance with Tukey's post hoc test). (B) Immunofluorescence results demonstrate the effect of PCB2 on the expression of NG2 (green) in the CC region of CPZ mice, as well as the average co‐localization area fraction (%) of GPX4 (red) in NG2+ (Magnification 40×, Scale Bar = 100 μm, $\bar{x}$ ± SD, *n* = 3). *p < 0.05, **p < 0.01, ***p < 0.001, comparison between Normal+PCB2 group and Normal group, comparison between other groups and CPZ + NS group (two‐way analysis of variance with Tukey's post hoc test). (C) Immunofluorescence results show the effect of PCB2 on the expression of GFAP (green) in the CC region of CPZ mice, and the average co‐localization area fraction (%) of GPX4 (red) in GFAP+ (Magnification 40×, Scale Bar = 100 μm, $\bar{x}$ ± SD, *n* = 3). *p < 0.05, **p < 0.01, ***p < 0.001, comparison between Normal + PCB2 group and Normal group, comparison between other groups and CPZ + NS group (two‐way analysis of variance with Tukey's post hoc test). (D) Immunofluorescence results illustrate the effect of PCB2 on the expression of IBA1 (green) in the CC region of CPZ mice, and the average co‐localization area fraction (%) of GPX4 (red) in IBA1+. (Magnification 40×, Scale Bar = 100 μm, $\bar{x}$ ± SD, *n* = 3). *p < 0.05, **p < 0.01, ***p < 0.001, comparison between Normal + PCB2 group and Normal group, comparison between other groups and CPZ + NS group (two‐way analysis of variance with Tukey's post hoc test). (E) Immunofluorescence staining was used to compare the average co‐localization area fraction (%) of Ferritin (green), NCOA4 (red), NRF2 (green), GPX4 (red) with MBP, NG2, GFAP, IBA1 in the CPZ + PCB2 group (Magnification 40×, Scale Bar = 100 μm, $\bar{x}$ ± SD, *n* = 3). *p < 0.05, **p < 0.01, ***p < 0.001, compared to the MBP group (two‐way analysis of variance with Tukey's post hoc test).

The results, as presented in Figure 3A–D, consistently show a reduction in the percentage of fluorescent co‐localization regions of MBP+, NG2+, GFAP+, and IBA1 + with GPX4 in the CPZ + NS group, compared to the control group (respectively, *p <* 0.01, *p <* 0.05, *p <* 0.01, *p <* 0.05), with a subsequent increase in the CPZ + PCB2 group (respectively, *p <* 0.05, *p <* 0.001, *p <* 0.01, *p <* 0.001), indicating PCB2's potential to modulate GPX4 expression. Figure 3A reveals a significant attenuation of MBP fluorescence in the CPZ+ NS group (*p <* 0.01), which was reversed in the CPZ + PCB2 group, suggesting a preservation of myelin integrity by PCB2 (*p <* 0.05). Figure 3B illustrates an upregulation of NG2 in the CPZ + NS group (*p <* 0.01), with an even greater increase in the CPZ + PCB2 group (*p <* 0.01), indicating enhanced oligodendrocyte progenitor cell activity. Figure 3C demonstrates an elevated GFAP fluorescence in the CPZ + NS group (*p <* 0.05), which was further increased with PCB2 treatment (*p <* 0.001), reflecting AS reactivity. Figure 3D shows a significant increase in Iba1 fluorescence in the CPZ + NS group (*p <* 0.01), and an amplified response in the CPZ + PCB2 group (*p <* 0.001), indicative of MG activation.

The collective immunofluorescence data suggest that PCB2 may exert neuroprotective effects by increasing GPX4 expression and modulating the expression of other proteins in various cell types within the CC region of CPZ mice. This upregulation could contribute to the mitigation of demyelination and oxidative stress, offering a potential therapeutic strategy for demyelinating diseases.

To understand the co‐localization of major glial cells in the CC region with Ferritin and GPX4 in the CPZ + PCB2 group, we conducted immunofluorescence co‐localization assays. We also included NCOA4, which plays a key role in the degradation of Ferritin, and NRF2, which regulates the expression of GPX4 (and also xCT) [21]. As shown in Figure 3E, among the four groups compared, we found that GPX4 colocalized most abundantly and visibly with the four main types of glial cells in the CC region. Moreover, in the GPX4 co‐localization, compared to the MBP group, the percentage of GPX4 fluorescence co‐localization area was significantly increased in the NG2, GFAP, and IBA1 groups (respectively, *p <* 0.05, *p <* 0.001, *p <* 0.001), indicating that PCB2 most prominently promotes the expression of GPX4 in AS in the CC region of CPZ mice.

### 3.5 PCB2 Increases the Expression of GPX4 and xCT in AS and the Content of GSH in the Myelin Debris of CPZ Mice

**FIGURE 4:**
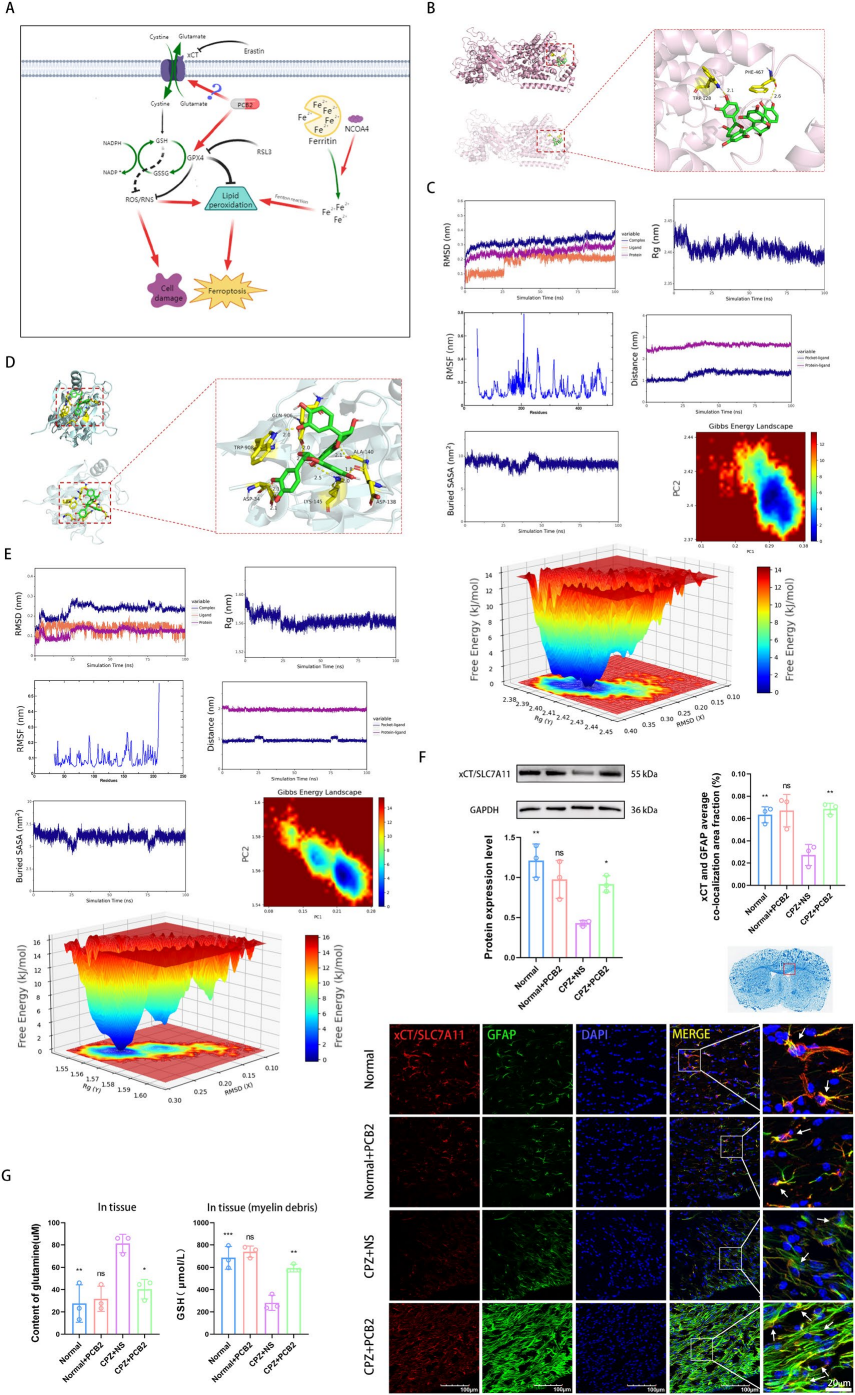
Analysis of molecular interactions in CPZ mice treated with PCB2 and its effects on the xCT/GSH/GPX4 axis in their AS. (A) Schematic Diagram of the Molecular Mechanism by Which PCB2 Regulates the xCT/GSH/GPX4 Axis to Inhibit Cell Damage and Ferroptosis. (B) PCB2 binds to the active pocket of the xCT/SLC7A11 protein on the surface, forming hydrogen bonds with the residues TRP‐128 and PHE‐467 of the SLC7A11 protein, with a binding energy of −1.96 (Kcal/mol). (C) Depicts the MD simulation results for the PCB2—SLC7A11 complex, covering stability—analysis and free—energy landscape to highlight thermodynamic—stability and ligand—protein interactions. (D) PCB2 binds to the active pocket of the GPX4 protein on the surface and forms hydrophobic interactions with the residues ASP‐34, TRP908/GLN‐906, etc., of the GPX4 protein, with a binding energy of −6.09 (Kcal/mol). (E) Illustrates the MD simulation findings for the PCB2—GPX4 complex. Stability—analysis and free—energy landscape are presented to underscore the thermodynamic—stability and ligand—protein interactions. (F) Western blot detection results show the effect of PCB2 on the protein expression level of xCT in the brain homogenate of CPZ mice ($\bar{x}$ ± SD, *n* = 3). Immunofluorescence staining results show the effect of PCB2 on the expression of xCT in the CC region of CPZ mice, as well as the co‐localization of xCT and GFAP (Magnification 40×, Scale Bar = 100 μm, $\bar{x}$ ± SD, *n* = 3). *p < 0.05, **p < 0.01, comparison between Normal + PCB2 group and Normal group, comparison between other groups and CPZ + NS group (two‐way analysis of variance with Tukey's post hoc test). (G) Enzyme assay detection shows the effect of PCB2 on the content of Glu in tissue cells in the CC region of CPZ mice, and increases the content of GSH in myelin debris ($\bar{x}$ ± SD, *n* = 3). *p < 0.05, **p < 0.01, ***p < 0.001, comparison between Normal + PCB2 group and Normal group, comparison between other groups and CPZ + NS group (two‐way analysis of variance with Tukey's post hoc test).

As shown in Figure 4A, PCB2 promoted GPX4 expression in AS in the brain of CPZ mice and increased the content of GSH in the brain (Figure 2A). The antioxidant and lipid peroxidation effects of GPX4 require GSH as an important co‐molecule, and xCT can affect the synthesis of GSH by regulating the input of Cys. Therefore, xCT also plays a key role in preventing cellular lipid peroxidation. We used molecular docking technology to simulate the docking of PCB2 with xCT and GPX4 (GSH is a tripeptide with a small molecular weight and cannot form residues for molecular docking visualization), confirming its possible spontaneous binding, as shown in Figure 4B,D.

In the MD study, the Gromacs2022 program was employed to conduct detailed analyses of the complexes between PCB2 and SLC7A11 protein (Figure 4C), as well as PCB2 and GPX4 protein (Figure 4E). By constructing simulation systems comprising small‐molecule ligands and proteins, and performing simulations under constant temperature, constant pressure, and periodic boundary conditions, the structural stability of the complexes and the binding characteristics between small molecules and proteins were explored.

The simulation results revealed that ligand‐protein complexes maintained structural stability throughout the simulation. Both RMSD and Rg analyses showed that the structure of the complex gradually stabilized, with the RMSD reaching equilibrium at around 0.3 nm after 25 ns. The RMSF analysis shed light on the varying flexibility across different protein regions. Furthermore, the analyses of centroid evolution and buried SASA indicated stable binding of the small molecule to the protein's binding site. Free‐energy landscape analysis further demonstrated that the complexes predominantly occupied lower‐free‐energy regions during the simulation, reflecting thermodynamic stability. Collectively, these findings provide crucial insights into the interaction between the small molecule and the protein, as well as the dynamic properties of the protein.

As shown in Figure 4F, the Western blot results showed that compared to the Normal group, the protein expression level of xCT in the CPZ + NS group was significantly reduced (*p <* 0.01). Compared to the CPZ + NS group, the protein expression level of xCT in the CPZ + PCB2 group was significantly increased (*p <* 0.05). This indicates that PCB2 regulates the protein expression of xCT. At the same time, the immunofluorescence staining results showed that compared to the Normal group, the average fluorescence intensity of co‐localization staining of GFAP and xCT in the CPZ + NS group was significantly reduced (*p <* 0.001). Compared to the CPZ + NS group, the average fluorescence intensity of co‐localization staining of GFAP and xCT in the CPZ + PCB2 group was significantly increased (*p <* 0.05).

Cys is the raw material for synthesizing GSH and antagonizes with Glu. Excessive release of Glu can inhibit the uptake of Cys by xCT, thereby inhibiting the synthesis of GSH. Therefore, the reduction of intracellular Glu can promote the synthesis of GSH. To further clarify the content of Glu and GSH in the myelin debris of the CPZ mouse, we detected the content of Glu in the CC region brain tissue. The results showed that compared with the Normal group, the content of Glu in the CPZ + NS group was significantly increased (*p <* 0.01). Compared with the CPZ + NS group, the content of Glu in the CPZ + PCB2 group was significantly reduced (*p <* 0.05). We then detected the content of GSH in the myelin debris. The results showed that compared with the Normal group, the content of GSH in the CPZ + NS group was significantly reduced (*p <* 0.001). Compared with the CPZ + NS group, the content of GSH in the CPZ + PCB2 group was significantly increased (*p <* 0.01), as shown in Figure 4G. This indicates that PCB2 promotes the protein expression of xCT in AS and regulates the content of GSH and Glu in the brain tissue cells. In this study, we measured the content of glutamine (rather than glutamate), as glutamine is a direct precursor of glutamate and their interconversion is crucial for cellular metabolism and function. The level of glutamine can reflect the metabolic status of cells and is closely related to the level of glutamate. Therefore, by measuring glutamine, we can indirectly infer the metabolism of glutamate and further understand the metabolic changes of cells in specific pathological states.

### 3.6 PCB2 Regulates the xCT/GSH/GPX4 Axis in RAs

**FIGURE 5:**
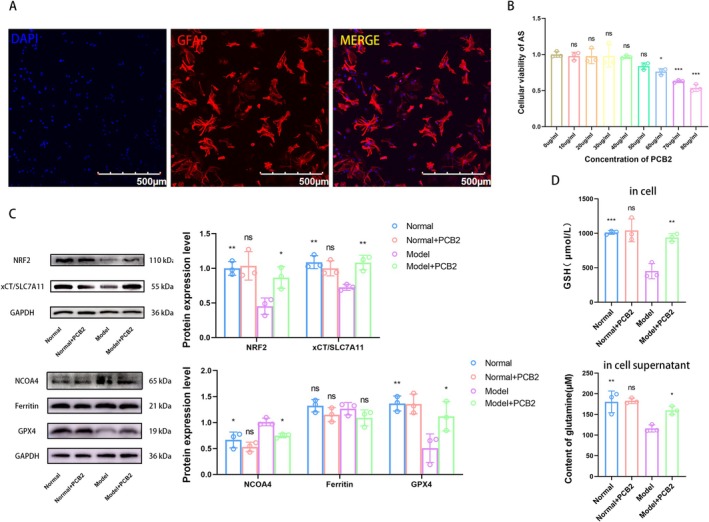
The effect of PCB2 on the xCT/GSH/GPX4 axis in RAs. (A) Immunofluorescence staining was used to assess the purity of primary AS, which was found to be greater than 95% (The scale bar = 500 μm). (B) The CCK‐8 assay identified the optimal concentration of PCB2 for AS as 50 μg/mL ($\bar{x}$ ± SD, *n* = 3). *p < 0.05, ***p < 0.001, compare with 0 μg/mL group (two‐way analysis of variance with Tukey's post hoc test). (C) Western blot analysis showed the effect of PCB2 on the protein expression levels of NRF2, xCT, Ferritin, and GPX4 in RAs ($\bar{x}$ ± SD, *n* = 3), *p < 0.05, **p < 0.01, comparison between Normal+PCB2 group and Normal group, comparison between other groups and Model group (two‐way analysis of variance with Tukey's post hoc test). (D) ELISA results indicated the effect of PCB2 on the levels of GSH within RAs and the concentration of Glu in the supernatant ($\bar{x}$ ± SD, *n* = 3). *p < 0.05, **p < 0.01, ***p < 0.001, comparison between Normal+PCB2 group and Normal group, comparison between other groups and Model group (two‐way analysis of variance with Tukey's post hoc test).

To determine whether PCB2 regulates the xCT/GSH/GPX4 axis in RAs, we proceeded with further exploration using a classic RAs model. We isolated AS from the brains of newborn mice and ensured the purity of the AS by immunofluorescence detection of the specific AS marker GFAP. The results showed that AS accounted for more than 95% of the total cell population, as shown in Figure 5A. We then used the CCK‐8 method to screen for the optimal concentration of PCB2 for AS, which showed that PCB2 had no effect on AS viability at concentrations below 50 μg/mL, as depicted in Figure 5B, providing a basis for determining the drug concentration for subsequent experiments.

Western blot analysis revealed that PCB2 significantly upregulated the expression of NRF2 and xCT in RA models compared to the non‐treated model (*p <* 0.05 and *p <* 0.01, respectively, Figure 5C). Despite a decrease in GPX4 in the model group (*p <* 0.01), PCB2 treatment significantly increased its expression (*p <* 0.05). Ferritin levels remained unchanged with PCB2 treatment, while NCOA4 increased in the model group (*p <* 0.05); PCB2 treatment significantly reduced its expression (*p <* 0.05).

Enzyme assays quantified GSH and Glu levels, showing a significant restoration of intracellular GSH (*p <* 0.01) and an increase in supernatant Glu (*p <* 0.05) in PCB2‐treated RAs groups compared to the model group (Figure 5D). These findings underscore the role of PCB2 in modulating the xCT/GSH/GPX4 axis in RAs.

### 3.7 PCB2 Alleviates Damage in RAs by Regulating the xCT/GSH/GPX4 Axis

**FIGURE 6:**
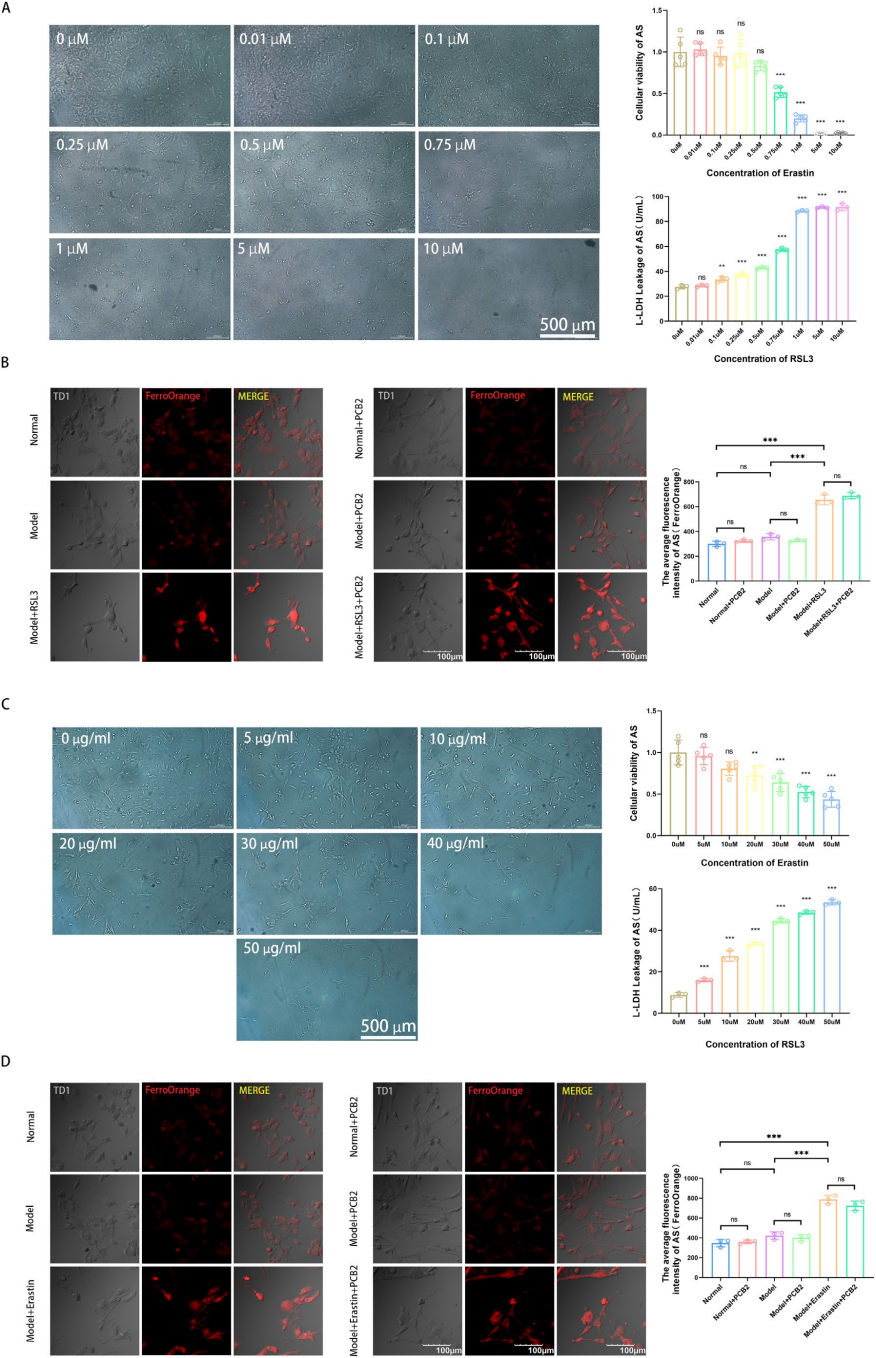
Screening of optimal concentrations of RSL3 and Erastin on AS and their effects on Fe^2+^ content within AS. (A) The Effect of Various Concentrations of RSL3 on the Morphology of AS Cells (The scale bar = 500 μm). CCK‐8 and L‐LDH assays identified the optimal concentration of RSL3 for AS cells as 0.75 μM ($\bar{x}$ ± SD, *n* = 5 for CCK‐8 and *n* = 3 for L‐LDH). **p < 0.01, ***p < 0.001, compare with 0 μM group (two‐way analysis of variance with Tukey's post hoc test). (B) FerroOrange Detection Results Show RSL3 Increased the Content of Fe^2+^ in RAs (The scale bar = 100 μm; $\bar{x}$ ± SD, *n* = 3), successfully Establishing the Model. ***p < 0.001 (two‐way analysis of variance with Tukey's post hoc test). (C) The Effect of Various Concentrations of Erastin on the Morphology of AS (The scale bar = 500 μm). CCK‐8 and L‐LDH assays identified the optimal concentration of Erastin for AS as 20 μM ($\bar{x}$ ± SD, *n* = 5 for CCK‐8 and *n* = 3 for L‐LDH). **p < 0.01, ***p < 0.001, compare with 0 μM group (two‐way analysis of variance with Tukey's post hoc test). (D) FerroOrange Detection Results Show Erastin Increased the Content of Fe^2+^ in RAs (The scale bar = 100 μm; $\bar{x}$ ± SD, *n* = 3), successfully Establishing the Model. ***p < 0.001 (two‐way analysis of variance with Tukey's post hoc test).

To determine whether PCB2 alleviates damage in RAs through the regulation of the xCT/GSH/GPX4 axis, we conducted rescue experiments using specific inhibitors. Initially, we used the CCK‐8 and L‐LDH assays to select the optimal cytotoxic concentration of the GPX4 inhibitor RSL3 for AS. The CCK‐8 and L‐LDH results showed that RSL3 began to cause significant shrinkage and death in AS at a concentration of 0.75 μM, with cell viability and cytotoxicity approaching 50% (Figure 6A), providing a basis for determining the drug concentration for subsequent experiments.

To clarify whether RSL3 induced ferroptosis in AS and the impact of PCB2, we used FerroOrange to detect the intracellular Fe^2+^ content (which is proportional to fluorescence intensity). The results showed no statistically significant difference in Fe^2+^ content between the Normal group and both the Model and Model + PCB2 groups (all *p >* 0.05); however, compared to the Normal group, the Fe^2+^ content was significantly enhanced in the Model + RSL3 group and the Model + RSL3 + PCB2 group (*p <* 0.001), as seen in Figure 6B. This indicates that RSL3 induced ferroptosis in AS.

Similarly, we used the CCK‐8 and L‐LDH assays once more to select the optimal cytotoxic concentration of the xCT inhibitor Erastin for AS. The CCK‐8 and L‐LDH results indicated that Erastin began to cause significant thinning and shrinkage of cellular processes and death in AS at a concentration of 20 μM, with cell viability and cytotoxicity approaching 50% (Figure 6C), providing a basis for determining the drug concentration for subsequent experiments.

To ascertain whether Erastin induced ferroptosis in AS and the impact of PCB2, we used FerroOrange to detect the intracellular Fe^2+^ content, which is proportional to fluorescence intensity. The results showed no statistically significant difference in Fe^2+^ content between the Normal group and both the Model and Model+PCB2 groups (all *p >* 0.05); however, compared to the Normal group, the Fe^2+^ content was significantly enhanced in the Model+Erastin group and the Model+Erastin+PCB2 group (*p <* 0.001), as depicted in Figure 6D. This suggests that Erastin induced ferroptosis in AS.

**FIGURE 7:**
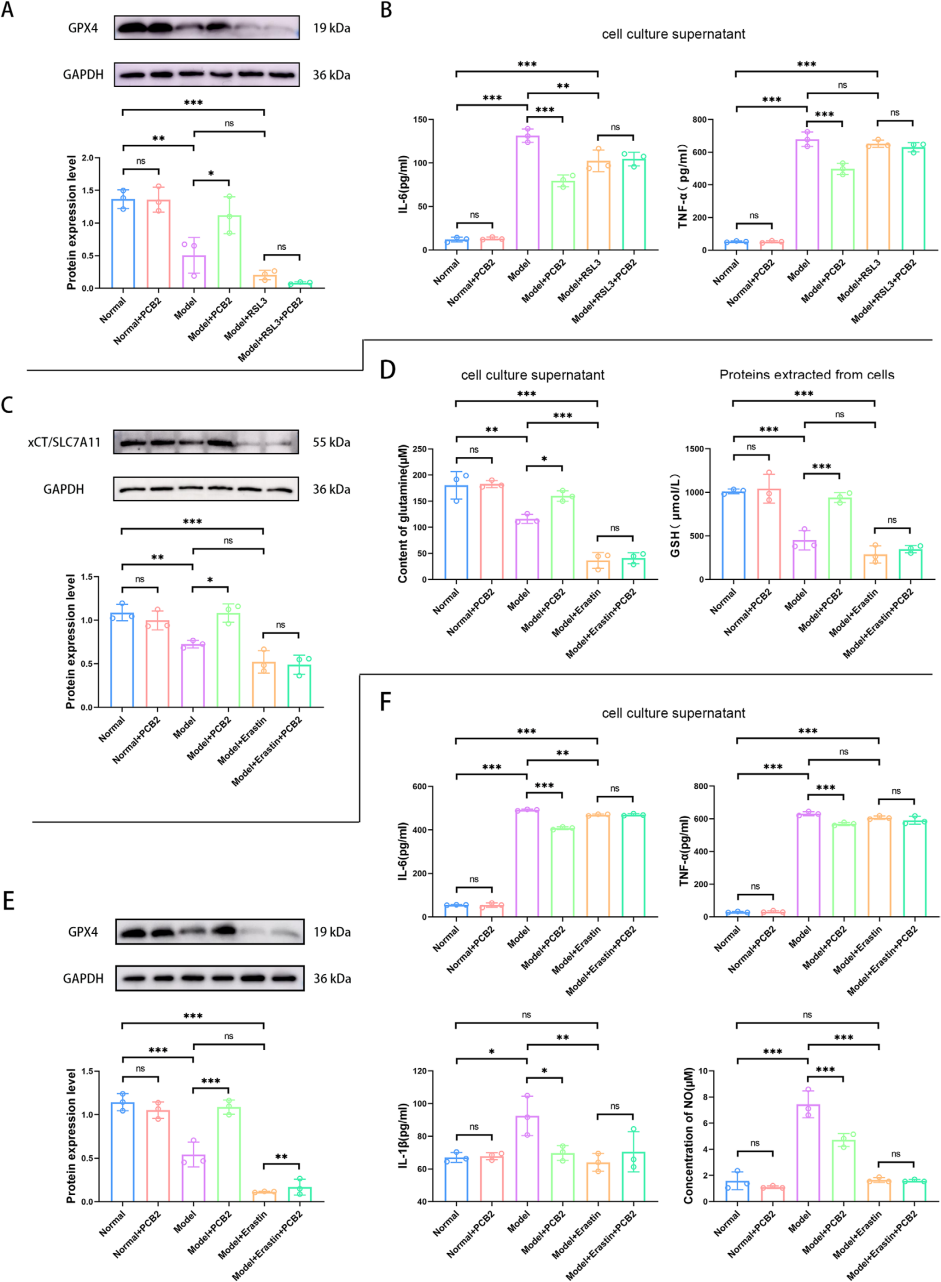
Exploring the molecular and cellular effects of PCB2 on xCT/GSH/GPX4 axis in RAs through cell recovery experiments. (A) Western blot analysis indicates the effect of PCB2 on the protein expression level of GPX4 in RAs ($\bar{x}$ ± SD, *n* = 3). *p < 0.05, **p < 0.01, ***p < 0.001 (two‐way analysis of variance with Tukey's post hoc test). (B) ELISA results show the effect of PCB2 on the content of TNF‐α and IL‐6 in the supernatant of RAs ($\bar{x}$ ± SD, *n* = 3). **p < 0.01, ***p < 0.001 (two‐way analysis of variance with Tukey's post hoc test). (C) Western blot analysis demonstrates the effect of PCB2 on the protein expression level of xCT in RAs ($\bar{x}$ ± SD, *n* = 3). *p < 0.05, **p < 0.01, ***p < 0.001 (two‐way analysis of variance with Tukey's post hoc test). (D) ELISA and enzyme assay results indicate the effect of PCB2 on the content of Glu in the supernatant of RAs and the content of GSH within the cells ($\bar{x}$ ± SD, *n* = 3). *p < 0.05, **p < 0.01, ***p < 0.001 (two‐way analysis of variance with Tukey's post hoc test). (E) Western blot analysis reveals the effect of PCB2 on the protein expression level of GPX4 in RAs ($\bar{x}$ ± SD, *n* = 3). **p < 0.01, ***p < 0.001 (two‐way analysis of variance with Tukey's post hoc test). (F) ELISA and enzyme assay results show the effect of PCB2 on the content of TNF‐α, IL‐6, IL‐1β, and NO in the supernatant of RAs ($\bar{x}$ ± SD, *n* = 3). *p < 0.05, **p < 0.01, ***p < 0.001 (two‐way analysis of variance with Tukey's post hoc test).

To determine the impact of PCB2 on the expression level of GPX4 protein in RAs after RSL3 treatment, we conducted a Western blot analysis. The results indicated that compared with the Model group, the GPX4 protein expression level was significantly increased in the Model+PCB2 group (*p <* 0.05). However, there was no statistically significant difference in the GPX4 protein expression level between the Model+RSL3 group and the Model+PCB2 group (both *p >* 0.05), as shown in Figure 7A. Subsequently, we further measured the content of TNF‐α and IL‐6 in the cell supernatant, which revealed that compared to the Model group, the levels of TNF‐α and IL‐6 in the Model+PCB2 group were significantly reduced (both *p <* 0.001). Compared with the Model group, there was no significant change in TNF‐α levels in the Model + RSL3 group (*p >* 0.05), while IL‐6 levels were significantly reduced (*p <* 0.01), and there was no significant difference in the levels of TNF‐α and IL‐6 between the Model+RSL3 group and the Model+RSL3 + PCB2 group (both *p >* 0.05), as depicted in Figure 7B. The combined results of Figure 7A,B suggest that when RSL3 inhibits GPX4, PCB2 has no significant effect on the state of RAs, indicating that PCB2 specifically alleviates the damage to RAs by regulating the GPX4 target.

Similarly, to ascertain the impact of PCB2 on the expression level of xCT protein in RAs after Erastin treatment, we conducted a Western blot analysis. The results showed that compared to the Model group, the xCT protein expression level was significantly increased in the Model+PCB2 group (*p <* 0.05). However, there was no statistically significant difference in the xCT protein expression level between the Model + Erastin group and the Model + Erastin+PCB2 group (both *p >* 0.05), as illustrated in Figure 7C. We then further measured the content of Glu in the cell supernatant and the content of GSH within the cells, which indicated that compared to the Model group, the content of Glu in the Model+PCB2 group was significantly increased (*p <* 0.05). Compared with the Model group, the Glu content in the Model + Erastin group was significantly reduced (*p <* 0.001); and there was no significant difference in the content of Glu between the Model + Erastin group and the Model+Erastin+PCB2 group (both *p >* 0.05). The detection of GSH content within AS revealed that compared to the Model group, the GSH content in the Model + PCB2 group was significantly increased (*p <* 0.001). Compared with the Model group, there was no significant change in GSH content in the Model + Erastin group (*p >* 0.05), but there was a trend of reduction; and there was no significant difference in GSH content between the Model+Erastin group and the Model+Erastin+PCB2 group (*p >* 0.05) as shown in Figure 7D.

After Erastin treatment, which reduced the protein expression level of xCT and the content of GSH in RAs, it would inevitably affect the downstream GPX4 protein expression. Therefore, we conducted a Western blot analysis, which showed that compared to the Model group, the GPX4 protein expression level in the Model+PCB2 group was significantly increased (*p <* 0.001). However, there was no statistically significant difference in the GPX4 protein expression level between the Model+Erastin group and the Model+Erastin+PCB2 group (both *p >* 0.05), as depicted in Figure 7E. Subsequently, we further measured the content of TNF‐α, IL‐6, IL‐1β, and NO in the cell supernatant, which revealed that compared to the Model group, the levels of TNF‐α, IL‐6, IL‐1β, and NO in the Model + PCB2 group were significantly reduced (respectively, *p <* 0.001, *p <* 0.001, *p <* 0.05, and *p <* 0.001). Compared with the Model group, there was no significant change in TNF‐α levels in the Model + Erastin group (*p >* 0.05), while IL‐6, IL‐1β, and NO were significantly reduced (respectively, *p <* 0.01, *p <* 0.01, *p <* 0.001); and there was no significant difference in the levels of TNF‐α, IL‐6, IL‐1β, and NO between the Model + Erastin group and the Model + Erastin+PCB2 group (both *p >* 0.05), as shown in Figure 7F.

### 3.8 Protective Effect of PCB2 on OLs via AS Axis Regulation

**FIGURE 8:**
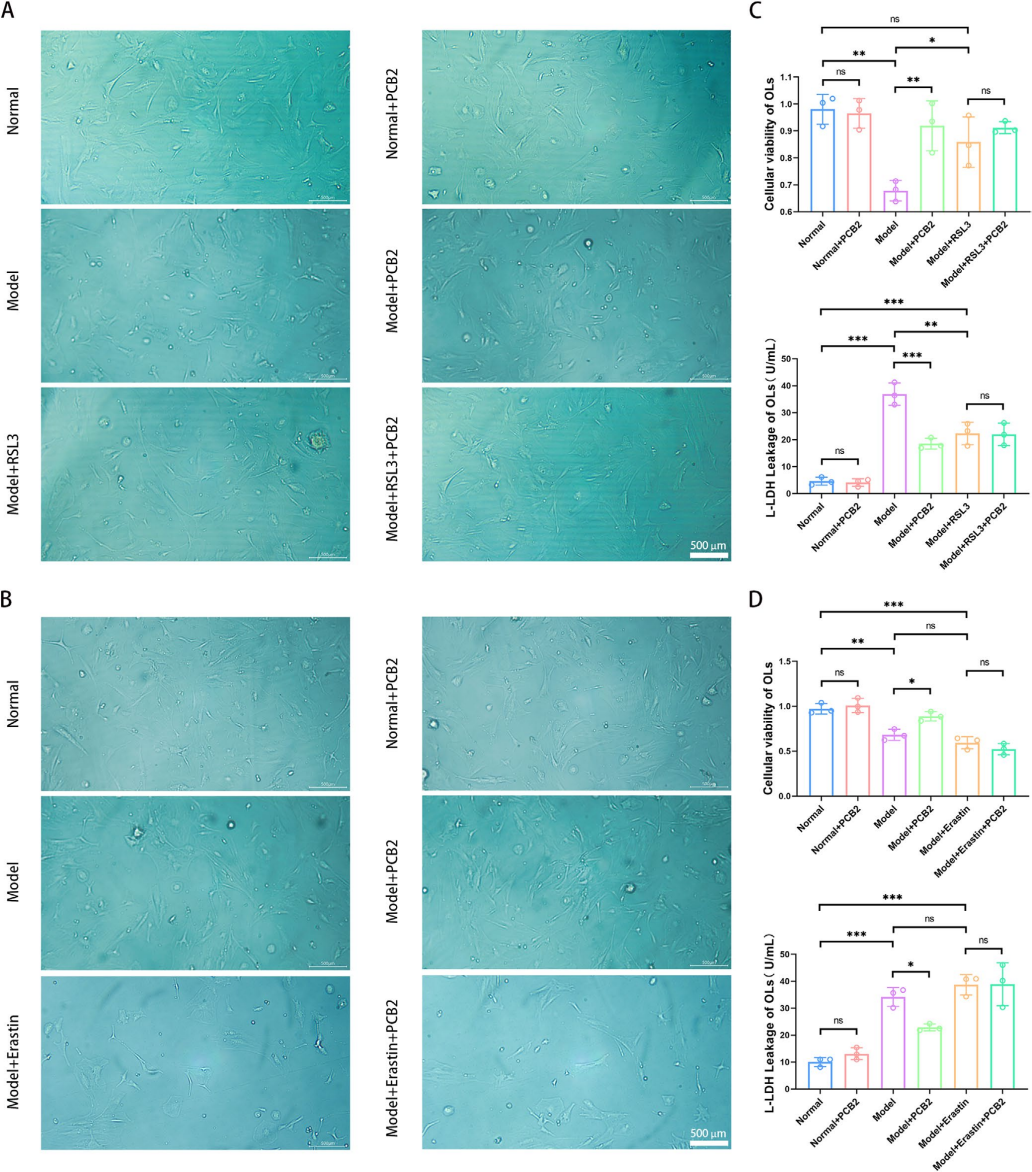
The effect of PCB2 on the morphology and survival rate of OLs by targeting RAs. (A) Representative microscope images showing the effect of PCB2 on OLs morphology and cell density in each group treated with conditioned medium from RAs pre‐treated with RSL3. Scale bar = 500 μm. (B) Representative microscope images showing the effect of PCB2 on OLs morphology and cell density in each group treated with conditioned medium from RAs pre‐treated with Erastin. Scale bar = 500 μm. (C) CCK‐8 and L‐LDH assay results show the effect of PCB2 on the cell viability and cytotoxicity of OLs in each group treated with the supernatant from RAs pre‐treated with RSL3 ($\bar{x}$ ± SD, *n* = 3). *p < 0.05, **p < 0.01, ***p < 0.001 (two‐way analysis of variance with Tukey's post hoc test). (D) CCK‐8 and L‐LDH assay results show the effect of PCB2 on the cell viability and cytotoxicity of OLs in each group treated with the supernatant from RAs pre‐treated with Erastin ($\bar{x}$ ± SD, *n* = 3). *p < 0.05, **p < 0.01, ***p < 0.001 (two‐way analysis of variance with Tukey's post hoc test).

As depicted in Figure 8A,C, compared to the model group, the model group treated with PCB2 showed increased cell density, shorter processes, and an improvement in cell viability with a decrease in cytotoxicity (respectively, *p <* 0.01 and *p <* 0.001). Compared with the model group, the model group treated with RSL3 showed an increase in cell viability with a corresponding decrease in cytotoxicity (respectively, *p <* 0.05 and *p <* 0.01);

No significant differences in cell viability or cytotoxicity were observed between the model group treated with RSL3 and the same group further treated with PCB2 (*p >* 0.05), indicating that the supernatant from RAs treated with PCB2 does not significantly affect OLs when GPX4 is inhibited by RSL3.

Similarly, as depicted in Figure 8B,D, in comparison with the model group, the model group treated with PCB2 exhibited increased cell density and improved cell viability with reduced cytotoxicity (both *p <* 0.05). Compared with the model group, the model group treated with Erastin showed no significant differences in cell viability or cytotoxicity. But there was a trend of decrease in cell viability with a corresponding increase in cytotoxicity (both *p >* 0.05); no significant differences in cell viability or cytotoxicity were found between the model group treated with Erastin and the same group further treated with PCB2 (*p >* 0.05), suggesting that the supernatant from RAs treated with PCB2 does not significantly influence OLs when xCT is inhibited by Erastin.

In summary, we infer that PCB2 can reduce cell damage in OLs by regulating the xCT/GSH/GPX4 axis in AS.

## 4 Discussion

In recent years, oxidative stress has garnered increasing attention in the pathogenesis of MS. Oxidative stress stems from an imbalance between the production and clearance of ROS and RNS within cells. Studies have shown that inflammatory responses and oxidative stress promote each other and are jointly involved in the pathological process of MS [22]. Oxidative stress can also affect the function of immune cells, promoting the occurrence and development of inflammatory responses. ROS produced during oxidative stress can increase the permeability of the BBB, allowing immune cells and inflammatory mediators to more easily enter the CNS. Excessive production of ROS and RNS can damage cellular DNA, proteins, and lipids, leading to cell injury and death. In this process, oxidative stress, as an imbalance between the production and clearance of ROS and RNS, leads to the accumulation of these reactive substances. These active substances can directly attack unsaturated fatty acids on the cell membrane, triggering lipid peroxidation and further causing cell ferroptosis. Therefore, we use PCB2, which has good antioxidant properties and the characteristic of protecting myelin sheaths, as the research subject for the treatment of this disease.

CPZ is a copper ion chelator, and its induced demyelination model is a classic animal research model for simulating MS demyelination and regeneration, with obvious pathological changes and characteristics of MS [23]. It is widely used to study central demyelination and myelin regeneration. Its neurotoxin can disrupt the copper homeostasis in the CNS, leading to pathological changes such as OLs damage, myelin degeneration and loss, inflammatory responses, oxidative stress, and lipid peroxidation. Furthermore, in the CPZ mouse model, cognitive and behavioral abnormalities such as memory decline, mania, and anxiety may emerge in later stages. Therefore, we assessed the impact of PCB2 on the behavioral profile of CPZ mice and found that the drug significantly improved cognitive function and alleviated symptoms of mania and anxiety. One thing to note is that, in our study, the CPZ + NS group exhibited increased total movement and central zone activity, which we interpreted as hyperactivity and anxiety. While this may seem contradictory to the classic interpretation of OFT, where reduced central zone exploration typically indicates increased anxiety, the CPZ model may induce a specific behavioral phenotype characterized by the coexistence of hyperactivity and anxiety [24]. Demyelination is a typical manifestation of MS onset, and MBP is a signature protein that constitutes myelin sheaths. In the CPZ model mice, the CC is the primary site of demyelination in the brain. We employed LFB, TrueGold, and immunofluorescence staining to assess demyelination in the CC region of acute CPZ demyelinated mice. Compared to the control group, the CPZ + NS group exhibited significant demyelination in the CC region, along with a loss of MBP expression and an increase in dMBP expression. PCB2 notably ameliorated these pathological conditions, indicating that it can inhibit demyelination in the CC region of the CPZ mouse model.

Subsequently, we conducted tests on the whole brains of CPZ mouse models and found that PCB2 could reduce the levels of IL‐1β, IL‐6, TNF‐α, NO, and LPO in the brain homogenates of CPZ mice, while increasing the levels of IL‐10 and GSH, as well as the activity of CAT, SOD, and GSH‐Px. This suggests that PCB2 exerts anti‐inflammatory, antioxidant, and anti‐lipid peroxidation effects in the CPZ mouse model. To further confirm that the effects of PCB2 also occur in the myelin debris, we extracted myelin debris from each group using an ultracentrifuge for analysis. The results showed that PCB2 could decrease the levels of LPO and MDA and increase the levels of GSH (Figure 4G) and the activity of CAT and SOD in the myelin debris of CPZ mice. This indicates that the antioxidant and anti‐lipid peroxidation capabilities of PCB2 also act in the myelin debris.

In the CPZ‐induced demyelination model, it has been proven that ferroptosis caused by lipid peroxidation is involved in the regulation of OL death in mice [25, 26]. Therefore, lipid peroxidation, as a key link in ferroptosis, is our focus of attention. Ferritin degradation has been identified as a critical step in promoting the occurrence of lipid peroxidation [27, 28, 29]. There is also a large amount of existing research evidence that GPX4 can be used as a reference sign to judge cell ferroptosis. The inactivation of GPX4 leads to the disruption of the oxidation balance, the accumulation of lipid peroxides, the destruction of the membrane structure of OLs, and ultimately leads to the ferroptosis of OLs [30].

Consequently, Ferritin and GPX4 can serve as sensitive indicators for detecting ferroptosis in cells. Additionally, NCOA4 facilitates the transport of Ferritin to autophagosomes by binding to it and subsequently fuses with lysosomes to degrade Ferritin and release iron [31]. We employed immunofluorescence to assess the expression of Ferritin, NCOA4, and GPX4 proteins in the CC region of the mouse brains across different groups, and found that the average co‐localization area ratio of Ferritin and NCOA4 was increased, while the expression of GPX4 protein was decreased in the CPZ mouse model; whereas PCB2 could reduce the average co‐localization area ratio of Ferritin and NCOA4 and increase the expression of GPX4 protein. Combined with the results from Western blot, this indicates that ferroptosis occurred in the CC region of CPZ mice, and PCB2 can inhibit ferroptosis in this area. When demyelination occurs in the CC region of CPZ mice, a large number of glial cells appear and participate in the process, including AS, MG, and oligodendrocyte precursor cells (OPCs), in addition to OLs. Our study found that PCB2 increased the protein expression (average co‐localization area ratio) of these four types of cells in the CC region of CPZ mice. To explore the relationship between the changes in Ferritin and GPX4 protein expression and these cells in the model + PCB2 group, we used immunofluorescence co‐localization assays and included NCOA4, which plays a key role in the degradation of Ferritin, and NRF2, which regulates the expression of GPX4 (NRF2 also regulates the expression of xCT). The results showed that the average co‐localization area of GPX4 with AS was the largest.

GSH is the core of amino acid metabolism abnormalities that occur in lipid peroxidation, and the depletion of GSH leads to a decrease in GPX4 activity. The cystine/glutamate antiporter (System Xc‐, xCT) is responsible for taking up extracellular Cys and releasing glutamate, and Cys and glutamate enter and exit the cell in a 1:1 ratio, maintaining a stable material exchange inside and outside the cell [32]. Cys is the raw material for synthesizing GSH, and the release of glutamate from xCT to the outside of the cell represents a reduction in the amount of Cys taken into the cell, which inhibits the synthesis of GSH, leading to the accumulation of downstream‐produced ROS, and ROS can directly act as an initiator of lipid peroxidation, causing the accumulation of lipid peroxides and leading to ferroptosis [33]. Therefore, the regulation of the xCT/GSH/GPX4 axis is crucial for reducing the lipid peroxidation damage of cells in the body.

We investigated whether PCB2 regulates the function or protein expression levels of xCT in A. We utilized molecular docking to verify the spontaneous binding potential of PCB2 with GPX4 and xCT. MD simulations suggest physical interaction between PCB2 and xCT/GPX4 active sites, supporting its role in functionally modulating their activity and increasing protein expression levels. Immunofluorescence staining confirmed that PCB2 increased the expression of xCT in AS of the CC region in CPZ mice (as measured by the average co‐localization area ratio). Enzyme assays demonstrated that PCB2 increased the levels of GSH and decreased those of Glu in AS. These results indicate that PCB2 regulates the entire xCT/GSH/GPX4 axis in AS.

Our findings demonstrate that the impact of PCB2 on the expression of GPX4 in AS within the CC region of the brain of CPZ mice is the most obvious, and moreover, PCB2 can also regulate the entire xCT/GSH/GPX4 axis of AS. RAs refer to the changes in morphology and function of AS when the CNS is damaged or pathologically stimulated, such as increased cell body, increased processes, and increased expression of GFAP. The activation and functional changes of AS are closely related to inflammatory reactions, neuroprotection, and neurorepair processes [34]. AS can differentiate into different phenotypes to perform different functions, such as A1‐type AS that can release pro‐inflammatory factors and neurotoxic molecules, which may cause neuronal damage. In some cases, they potentially exacerbate nerve system damage and disease progression by releasing harmful substances or over‐activation, and studies have shown that their conditional culture fluid can significantly damage the differentiation ability of OLs and inhibit myelin regeneration [35]. However, the role of RAs may also be beneficial; A2‐type AS can release anti‐inflammatory factors and neurotrophic factors, supporting neuronal survival and functional recovery [36]. RAs play an important role in the repair of CNS damage, neuroinflammation, neurodegenerative diseases, etc. Studies have shown that RAs can promote the self‐renewal of OPCs [37]. AS is activated after MS lesions, releasing inflammatory mediators and cytokines such as IL‐1β and TNF‐α, participating in inflammatory reactions, and can also produce neurotrophic factors such as nerve growth factor (NGF) to support the survival and differentiation of OLs. Therefore, further exploration of targeted therapies for AS is expected to become a new target for the treatment of MS.

Next, we used the classic RAs model for in vitro experimental validation. We utilized western blot to demonstrate that PCB2 could increase the protein expression levels of NRF2, xCT, and GPX4 in RAs. The impact on Ferritin protein expression level was not statistically significant, but it decreased the protein expression level of NCOA4. Although the results in Figure 5C and Figure 6B,D indicate that PCB2 has no effect on Ferritin and iron content levels in AS, assessing the protein expression level of NCOA4 in RAs is useful, as it provides an indirect indicator of Ferritin degradation and iron release. Studies have shown that in CPZ‐induced demyelination models, despite no changes in Ferritin and iron content, changes in NCOA4 are also associated with ferroptosis [29]. Additionally, PCB2 increased the content of GSH in RAs and also increased the content of Glu in the cell supernatant, indicating that PCB2 enhanced the outward transport function of Glu in RAs.

In the recovery experiment, we utilized RAs and inhibitors RSL3 and Erastin as our research models. The results indicated that, compared to the model with inhibitors, there was no statistically significant difference after PCB2 treatment, which aligns with our expectation and suggests that PCB2 exerts its effects by modulating the xCT/GSH/GPX4 axis in AS. However, when comparing the model group to the model with inhibitors group, the latter showed equivalent or even lower levels of TNF‐α, IL‐6, IL‐1β, and NO, which were unexpected. This could be attributed to the significant reduction in the number of surviving cells in the model plus inhibitor group compared to the model and normal groups when we washed the well plate with PBS and changed the culture medium to prepare the 24–48 h cell supernatant. Based on the inhibitor concentration screening results in Figure 6A,C, cell viability was approximately 50% of that at a 0 μM concentration after inhibitor treatment. Consequently, if the contents of the aforementioned indicators are divided by the number of surviving cells, the results might indicate that the model with inhibitors group has higher values than the model group.

To clarify the effect of AS on OLs following PCB2 intervention, we designed an experiment co‐culturing RAs‐conditioned medium with OLs and demonstrated that PCB2 can specifically reduce OLs damage by modulating the xCT/GSH/GPX4 axis in RAs (Figure 8). The reason why the model with inhibitors group did not perform worse, and even better than the model group, might be the same as the reason mentioned above.

This study has limitations: firstly, while molecular docking and dynamics simulations suggest PCB2 binds to xCT and GPX4, functional validation via co‐immunoprecipitation or pull‐down assays remains necessary to confirm binding specificity in cellular contexts. Additionally, although NRF2 involvement in mediating PCB2's regulation of the xCT/GSH/GPX4 axis is inferred, its precise role remains unvalidated by knockout or overexpression models.

Despite these gaps, our study provides novel evidence that PCB2 mitigates CPZ‐induced oligodendrocyte damage and demyelination by regulating the xCT/GSH/GPX4 axis in astrocytes and suppressing lipid peroxidation. Collectively, these findings support the therapeutic potential of natural compounds targeting astrocytes for multiple sclerosis. Future investigations should explore transcriptional mechanisms, perform dose–response analyses, and clarify NRF2‐dependent pathways to fully elucidate PCB2's modulation of the xCT/GSH/GPX4 axis.

## 5 Conclusion

In conclusion, our study suggests that PCB2 can ameliorate the behavioral performance in CPZ mice, reduce inflammation, oxidative stress, lipid peroxidation, and damage to OLs, and inhibit demyelination. These effects may be associated with PCB2's regulation on the xCT/GSH/GPX4 axis in AS. Targeted modulation of AS function could represent a novel therapeutic strategy for demyelinating diseases. Our findings provide a theoretical foundation and new perspectives for the treatment of MS and the potential application of PCB2.

## Author Contributions

Jian Liu, Qing Wang, and Cun‐Gen Ma conceptualized and designed the research. Jian Liu, Yan‐Xia Hou, Meng Pu, and Lu‐Lu Zheng carried out the experiments. Zi‐Wei Zhang provided the materials. Ying Xiao analyzed the data. Jian Liu, Ya‐Jie Liang, Ying Chen, and Zhen Mao drafted the manuscript. All authors contributed through constructive discussions and expertise to the final version of the manuscript. The authors read and approved the published version.

## Ethics Statement

All applicable institutional and national guidelines for the care and use of animals were followed. The ethical review board of Shanxi University of Chinese Medicine granted approval for all experimental procedures (Docket # 2019LL106). This article does not contain any studies with human participants performed by any of the authors.

## Consent

The authors have nothing to report.

## Conflicts of Interest

The authors declare no conflicts of interest.

## Supporting information


**Appendix S1:** cns70598‐sup‐0001‐AppendixS1.pdf.

## Data Availability

All data used in this work can be acquired from the corresponding author upon reasonable request.
