# Supplementary material for: Proanthocyanidin B2 Alleviates Cuprizone‐Induced Demyelination by Regulating the Astrocytic xCT/GSH/GPX4 Axis
Source: CNS Neurosci Ther. 2025 Sep 18;31(9):e70598. doi: 10.1111/cns.70598 (PMC12446574; doi:10.1111/cns.70598)

Lane arrangements in all Western blot images are as follows:

For images with **four lanes**: from left to right, the lanes correspond to Normal group, Normal + PCB2 group, Model group, and Model + PCB2 group, respectively.

For images with **six lanes**: from left to right, the lanes are Normal group, Normal + PCB2 group, Model group, Model + PCB2 group, Model + inhibitor (RSL3/Erastin) group, and Model + inhibitor (RSL3/Erastin) + PCB2 group, in sequence.

Fig 2 C

NRF2 (100 kDa)

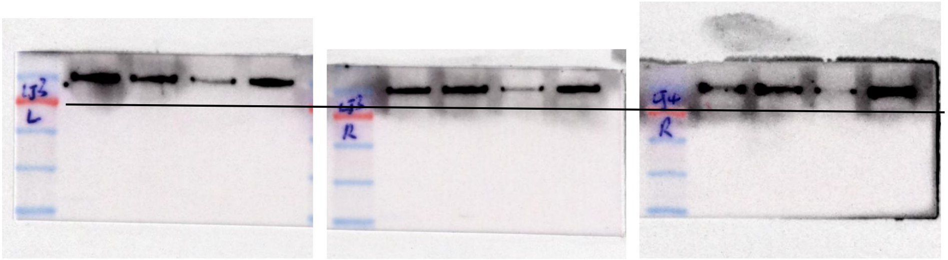

NCOA4 (70 kDa)

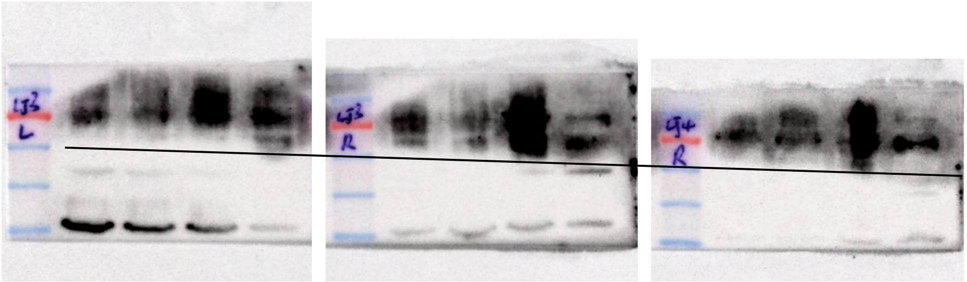

GAPDH (36 kDa)

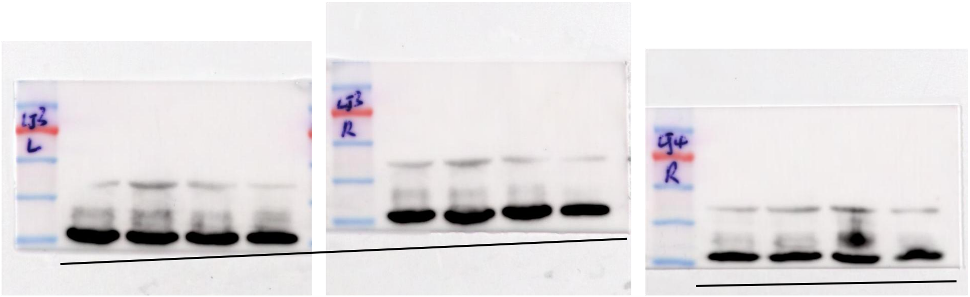

FERRITIN (21 kDa)

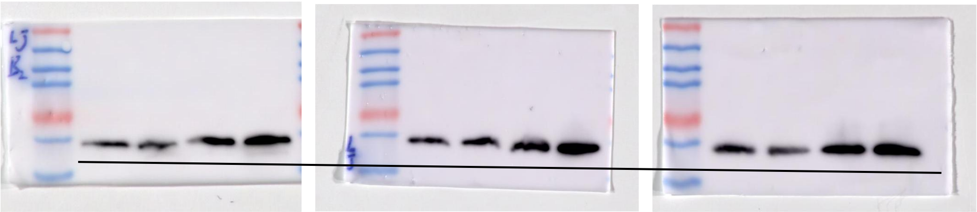

GPX4 (19 kDa)

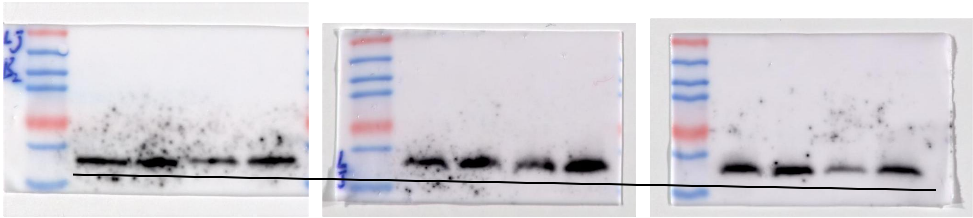

TUBULIN (55 kDa)

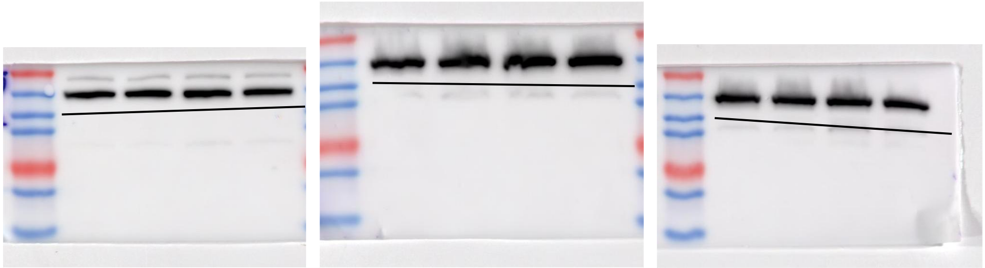

Fig 4 D

xCT/SLC7A11 (55 kDa)

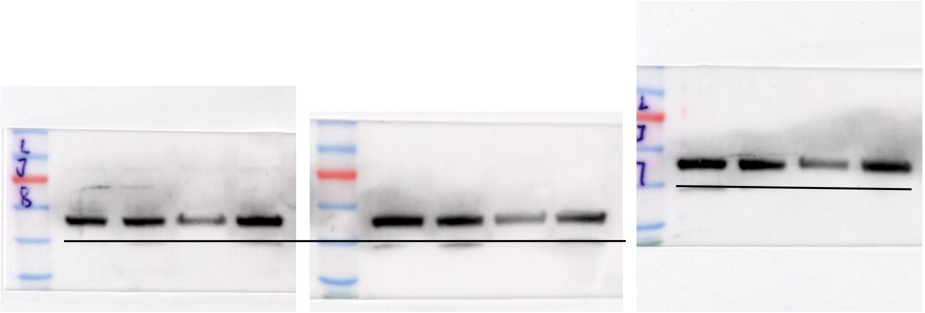

GAPDH (36 kDa)

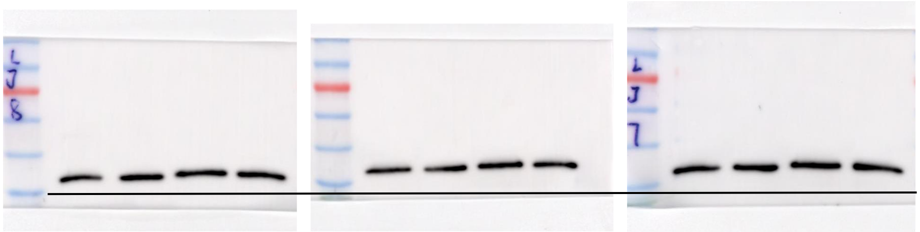

Fig 5 C

NRF2 (100 kDa)

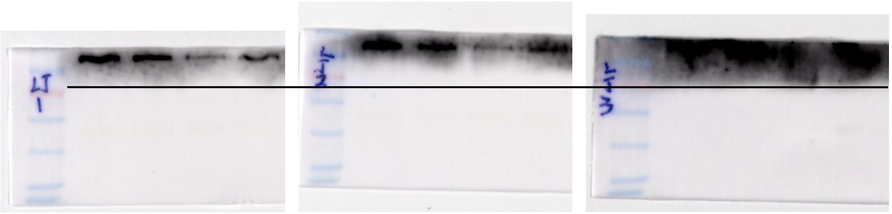

xCT/SLC7A11 (55 kDa)

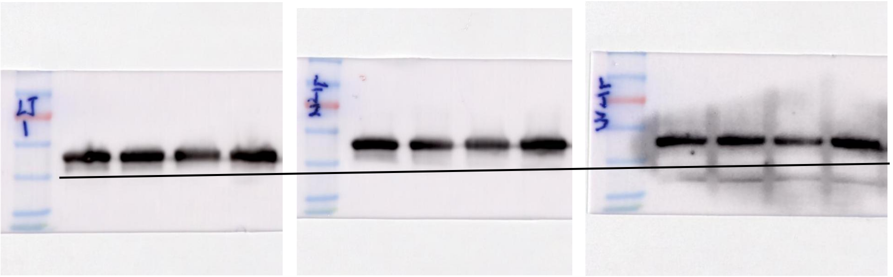

GAPDH (36 kDa)

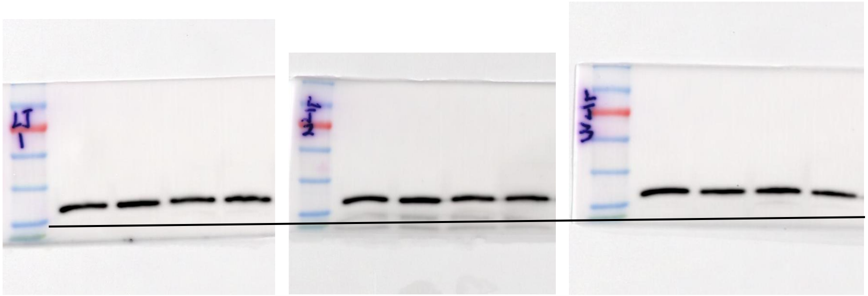

NCOA4 (70 kDa)

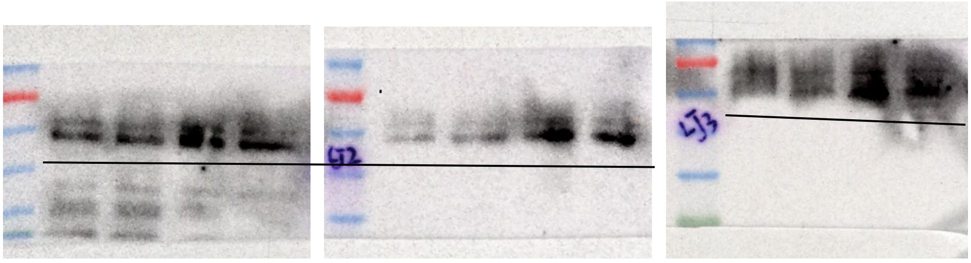

GAPDH (36 kDa)

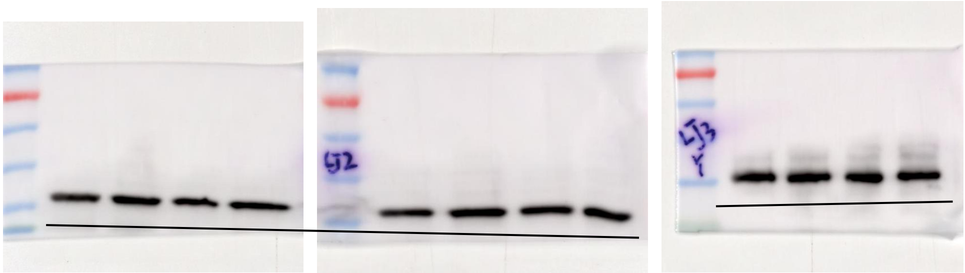

FERRITIN (21 kDa)

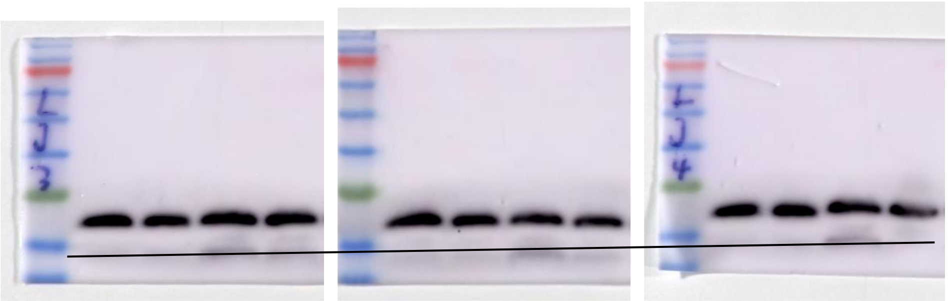

GPX4 (21 kDa)

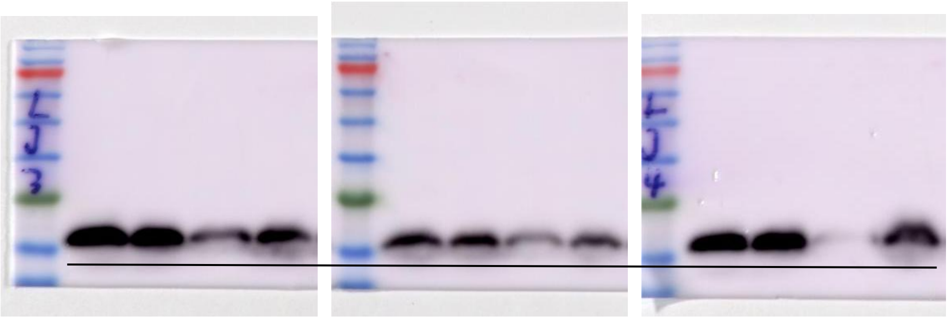

GAPDH (36 kDa)

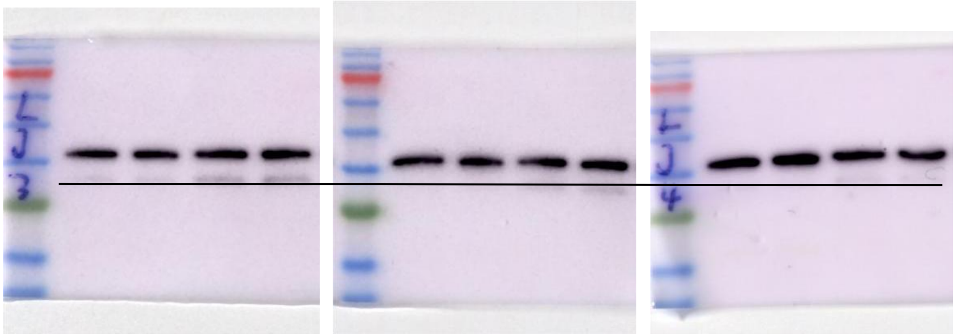

Fig 7

A:GPX4 (19 kDa)

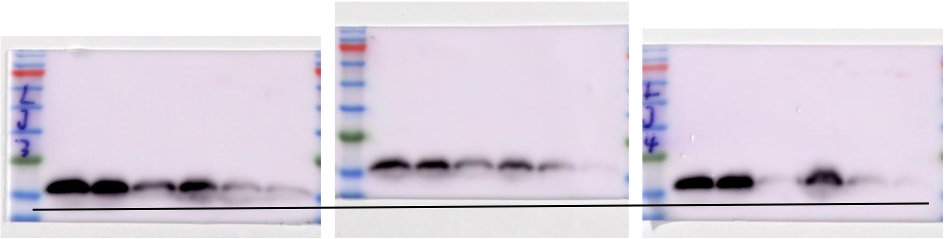

GAPDH (36 kDa)

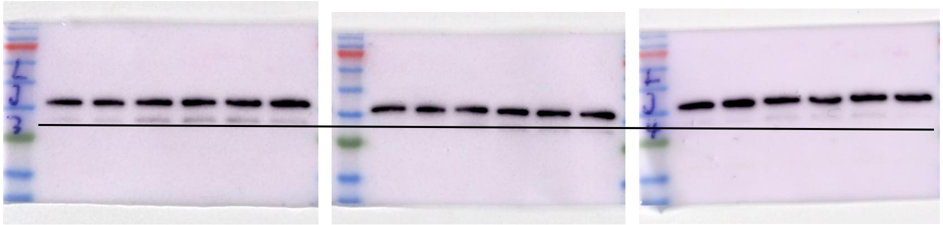

C:xCT/SLC7A11 (55 kDa)

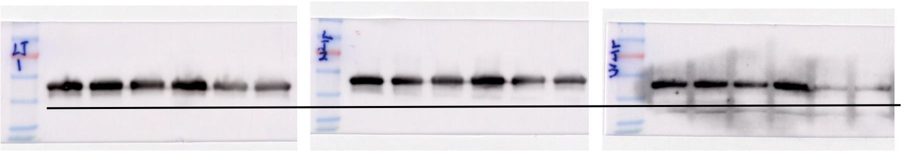

GAPDH (36 kDa)

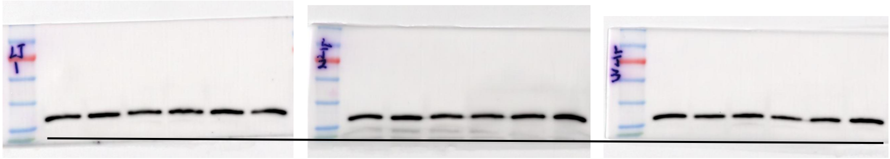

E:GPX4 (19 kDa)

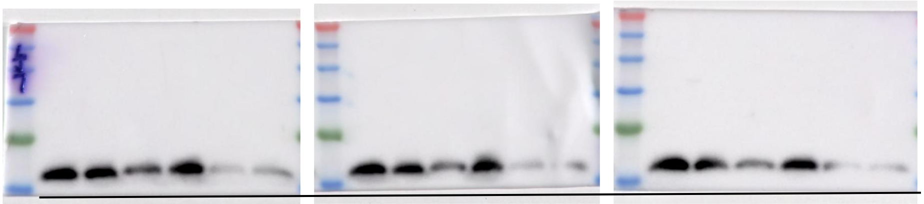

GAPDH (36 kDa)

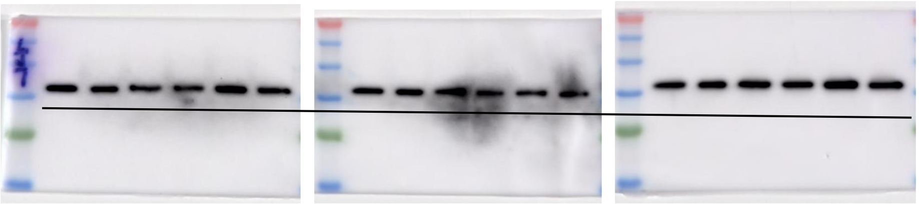

Supplement: Supplementary file 1 — Appendix S1: cns70598‐sup‐0001‐AppendixS1.pdf. [file CNS-31-e70598-s001.pdf]
